# A meta-analysis of the effects of mindfulness meditation training on self-reported interoception

**DOI:** 10.1038/s41598-025-22661-4

**Published:** 2025-11-06

**Authors:** Isaac N. Treves, Ya-Yun Chen, Caitlyn L. Wilson, Charles Verdonk, Joanne Qina`au, James E. Pustejovsky, Simon B. Goldberg, Wolf Mehling, Zev Schuman-Olivier, Sahib S. Khalsa

**Affiliations:** 1 Department of Psychiatry, New York State Psychiatric Institute, Columbia University Irving Medical Center, New York, 10032 USA; 2 Psychology, Virginia Tech, Blacksburg, 24061 USA; 3 Department of Psychology, University of Colorado Denver, Denver, 80204 USA; 4 French Armed Forces Biomedical Research Institute, Brétigny-sur-Orge, France; 5 UMR VIFASOM, Université de Paris, Paris, France; 6 Laureate Institute for Brain Research, Tulsa, OK 74136 USA; 7 Osher Center for Integrative Health, University of California, San Francisco, 94115 USA; 8 Educational Psychology Department, University of Wisconsin-Madison, Madison, WI 53706 USA; 9 Counseling Psychology, University of Wisconsin-Madison, Madison, WI 53706 USA; 10 Center for Mindfulness and Compassion, Department of Psychiatry, Cambridge Health Alliance, Cambridge, MA 02139 USA; 11 Department of Psychiatry, Harvard Medical School, Cambridge, MA 02139 USA; 12 Department of Psychiatry and Biobehavioral Sciences, Semel Institute for Neuroscience and Human Behavior, David Geffen School of Medicine, University of California at Los Angeles, 760 Westwood Plaza, Los Angeles, CA 90024 USA

**Keywords:** Mindfulness, Interoception, MAIA, Body awareness, Meditation, Meta-analysis, Human behaviour, Translational research

## Abstract

Mindfulness meditation training may cultivate interoceptive awareness and provide therapeutic benefit when implemented within mental and physical health interventions. This pre-registered meta-analysis evaluated the impact of mindfulness interventions on self-reported interoception measures and associated relationships with psychological outcomes. Twenty-nine randomized controlled trials with 2,191 participants (77.8% female, mean age 32.8 years) were meta-analyzed using correlated and hierarchical effects models. Interventions included mindfulness-based programs (*k* = 15), body-based approaches (incorporating elements like massage, *k* = 8), and other variations (*k* = 6). Five SIMs were tested; the Multidimensional Assessment of Interoceptive Awareness was the most common (22 studies). Results showed a small-to-medium positive effect on interoception measures across all studies (*g* = 0.31, *p* < 0.001, 95% CI [0.21, 0.42]) with low-to-moderate heterogeneity (*τ* = 0.16). Mindfulness-based programs demonstrated the largest effects (*g* = 0.41). No evidence of publication bias was found. No other moderators, such as practice dosage or clinical sample, were significant. Improvements in self-reported interoception were similar in size to improvements in self-reported mindfulness and were related to improvements in psychological distress. These meta-analytic findings provide evidence that mindfulness-based interventions lead to adaptive changes in the subjective experience of interoception, perhaps contributing to improved mental wellbeing.

**Supplementary Information:**

The online version contains supplementary material available at 10.1038/s41598-025-22661-4.

## Introduction

Interoception refers broadly to the processing of internal bodily signals by the nervous system^1^, and is related to emotional awareness, self-regulation, mental health, and cognition^1–4^. Interoception can be measured objectively, for example, by testing how accurately one can sense one’s resting heartbeat (interoceptive accuracy;^5,6)^, or via applied perturbations of physiological states^7^. Reduced interoceptive accuracy has been suggested to underly behavioral health difficulties in autism and other neurodevelopmental populations^8^, and is implicated in anxiety and depression^9–11^. Measuring interoception also frequently involves the collection of subjective components including interoceptive sensibility, attention, interpretation, and regulation of bodily signals. Subjective components of interoception are especially relevant in clinical contexts, where the use of self-report questionnaires often reflects a standardized effort to delineate symptoms and track symptom changes following treatment. Decreased levels of self-reported interoceptive attention and regulation are found in individuals with abnormalities of emotional experience, such as alexithymia^12,13^. Interoceptive catastrophizing (i.e., exaggerated symptom reporting) is also common in individuals with somatic symptom disorder, generalized anxiety disorder, and panic disorder^14^. Similar reports have been observed in other conditions including post-traumatic stress disorder, irritable bowel syndrome, fibromyalgia, eating disorders, and substance use disorders, underscoring the ubiquitous presence of abnormal interoception across a spectrum of health challenges^15^. Given the evidence that difficulties with interoception are related to psychopathology, there is substantial interest in cultivating interoceptive awareness through mind-body interventions^15–19^, including those utilizing mindfulness meditation^20,21^.

Mindfulness is often defined as a non-judgmental awareness of the present moment^22^, although this reflects a secular distillation from a broader historical Buddhist tradition. Mindfulness-Based Stress Reduction (MBSR)^23^ emerged as a paradigmatic program for training mindfulness in the context of efforts to achieve symptom reduction in various physical and mental conditions. Following standard recommendations^24^ we distinguish trainings based in theory and curricula of MBSR as mindfulness-based programs (MBP) from the more general health-based approaches, which we refer to as mindfulness-based interventions (MBI)^25^. MBSR consists of 8 weeks of mindfulness training through focused attention meditation, body scan meditation, mindful movement (e.g., yoga asana), dialogue and inquiry, and didactic instruction. While cognitive explanations of mindfulness emphasizing attention and emotion regulation predominate in the literature (e.g.,^26^), MBSR was initially conceived for individuals with chronic pain^27^, and transforming the process of sensing and interpreting body signals was always a central part of the therapeutic approach^28^. Many elements of MBSR involve body-focused awareness and a sustained top-down attentional focus on interoceptive signals^29^. For example, the most commonly taught focused attention meditation includes the practice of continuously attending to signals of the breath and discerning its movements throughout the nasal cavities, chest, diaphragm, and abdomen. Body scans involve shifting attention throughout the body and observing spontaneously arising signals. Mindful movement involves proprioceptive attention as the body changes position and orientation as well as breath awareness. All of these practices are taught with an emphasis on acceptance, present-moment sensory awareness, non-judgement and self-compassion, and formal and informal practice of these qualities are emphasized in didactics.

There is systematic meta-analytic evidence that mindfulness-based interventions involve improvements in certain objective measures of body awareness^30^. In that study, we advanced the framework of ‘accurate and correct monitoring’ to identify and categorized body awareness tasks, to include both ‘proximal’ tasks (e.g., heartbeat perception) and ‘distal’ tasks (e.g., monitoring emotional arousal in the body during a movie^31^). A meta-analysis with updated studies confirmed a small, heterogeneous effect of mindfulness on objective accuracy, that was most prominent for distal measures^32^. There was no evidence of differences in proximal tasks, such as heartbeat perception, as we have found previously^30,33^. These dissociations between proximal and distal measures may, in part, reflect process-specific practice effects, as most MBPs do not specifically target heartbeat perception. To date there are few MBPs evaluating a training effect on respiratory interoception.

While there are many studies examining the subjective aspects of interoception, there is currently no meta-analytic clarity about the effects of mindfulness or MBPs on self-reported interoception broadly. Several well-established self-reported interoception measures (SIM) exist. These include the Scale of Body Connection^34^, and the Multidimensional Assessment of Interoceptive Awareness^35,36^, but they have yet to be meta-analytically examined in the context of mindfulness. One open question is whether all such measures are equally sensitive to mindfulness meditation training. It seems plausible that differentiating these scales, including the distinguishing between maladaptive and adaptive subjective interoception^12,37^, may inform the understanding of processes involved in MBIs. To that end, a meta-analytic framework is useful for assessing statistical differences between scales. Other questions concern the relationships between improvements in interoception and improvements in distress and mindfulness. For example, a recent study found that interoceptive appreciation (measured by combining the MAIA Body Listen and Trusting subscales) partially mediated the effects of a mindfulness-based program on enhancing the capacity for health behavior change^38^. If relationships between changes in clinical outcomes and interoception are consistent across studies, this could support measurement of interoception as a process in MBIs, as well as mindfulness didactics focusing on interoceptive awareness^39^.

Previous studies have examined the effects of mind-body interventions on interoception in psychiatric disorders^15,18^, but a systematic interrogation of effects on SIMs is lacking. In the current study, we meta-analytically examined evidence from randomized control trials evaluating the effects of mindfulness interventions on self-reported interoception, including both MBP interventions structured similarly to MBSR, but also incorporating any interventions with teacher-led mindfulness meditation. Given some uncertainty about the scope of SIMs, we use measures incorporated previously in a construct review^40^, and we use modern meta-analytic techniques to account for correlated measures within studies^41^. We analyzed moderators including study adherence, study populations and practice quantities to inform theories of interoceptive skill acquisition. Our preregistered questions were:

(RQ1): Do mindfulness interventions improve self-reported interoception? If so, which constructs of interoception (e.g. adaptive or maladaptive) are influenced by mindfulness interventions?

(RQ2) What characteristics of mindfulness interventions and participant samples moderate effects on self-reported interoception?

(RQ3) Do changes in interoception relate to changes in psychological symptoms?

## Methods

### Objectives

The objective of this meta-analysis was to (RQ1) analyze the effects of mindfulness training on self-reported interoception, (RQ2) test moderation effects, and (RQ3) assess the relationship between changes in interoception, changes in psychological distress, and changes in mindfulness.

### Protocol and registration

The meta-analysis was preregistered at https://osf.io/yr439. Several deviations were made. Namely, we used expert input for dimensions of interoception instead of empirical factor analyses, given incomplete coverage of the present measures in empirical factor analyses^40^. In addition, we renamed ‘interoception’ interventions to ‘body-based’ interventions given that they included interoceptive, proprioceptive and body awareness practices generally. Most importantly, we found inadequate performance of the bivariate meta-analytic models for assessing relationships between changes in distress, mindfulness, and interoception. The models uniformly produced high correlation values, but also extremely large confidence intervals that varied based on model assumptions. For instance, the relationship between mindfulness and distress was significant assuming no sampling covariance, but when positing a moderate sampling covariance it was not significant. For this reason, we characterized the relationships using correlation coefficients and scatterplots but did not test statistical significance. We followed PRISMA guidelines^42^(Supplement 2: CHECKLIST).

### Eligibility criteria

We included only randomized controlled trials of MBIs that involved sustained (> 4 formal hours) practice of mindfulness meditation, defined as practices including: open awareness, breath meditation and body scans, with the focus of present-moment awareness and acceptance^43–45^. Interventions could also include other components such as yoga, walking and massage, as long as they were paired with mindfulness meditation. This decision was made to maintain a pluralistic and inclusive focus of MBIs^25,46^; however, we coded the relevant distinctions between interventions so as to isolate differential effects. Specifically, we categorized interventions as (1) MBPs (i.e., MBSR, MBCT, and other programs with the same training standards and curricular elements^24^, (2) body-based MBIs that included massage, and (3) other MBIs. We excluded app-based interventions given heterogeneous training durations, more limited adherence, and lack of teacher involvement^47^. See Supplemental Table S1 for a full list of included studies.

For the present meta-analysis, we refer broadly to interoception as the processing of internal bodily signals by the nervous system, and to interoceptive self-report as measuring interoceptive beliefs, attitudes, and thoughts. Studies in the current review must have included data from a questionnaire primarily developed for studying the construct of interoception. We specifically included any questionnaires that were present in a systematic review of self-report interoception constructs^40^ (Table 1). We additionally added two questionnaires – the Scale of Body Connection (SBC)^34^ and the Cardiac Anxiety Questionnaire (CAQ)^48^, because examination of their item-level responses revealed compatibility with the measurement of self-reported interoception. It should be noted that the Five-Facet Mindfulness Questionnaire and its ‘Observing’ subscale was not considered a self-report measure of interoception, as the item-level responses primarily assess awareness of external sensory experiences (e.g. sun on face, wind in hair) and general emotional awareness, rather than explicit perceptions of internal bodily signals such as respiration, heartbeat or visceral sensations, which are central to self-reported interoception. We only included empirical studies, allowing for preprints, theses, or book chapters, regardless of peer-review publication status. We allowed for all ages and populations of participants.

**Table 1:** Self-reported measures, along with their corresponding interoceptive dimensions and adaptive/maladaptive categorization.

Self-reported measures	Interoceptive dimension	Category
MAIA-NO, BAQ, SBC-A	Sensing	Adaptive
MAIA-ND, MAIA-SR, MAIA-AR,	Attention	Adaptive
MAIA-NW, MAIA-BL, MAIA-EA, MAIA-TR	Interpretation	Adaptive
VSI, CAQ	Sensing	Maladaptive
VSI, CAQ, SBC-D	Attention	Maladaptive
VSI, CAQ	Interpretation	Maladaptive

All SIMS reported are included. MAIA: Multidimensional Assessment of Interoceptive Awareness; MAIA-NO: Noticing subscale of the MAIA; BAQ: Body Awareness Questionnaire; SBC: Scale of Body Connection; SBC-A: Awareness subscale of the SBC; MAIA-ND: Not Distracting subscale of the MAIA; MAIA-SR: Self-Regulation subscale of the MAIA; MAIA-AR: Attention Regulation subscale of the MAIA; MAIA-NW: Not Worrying subscale of the MAIA; MAIA-BL: Body Listening subscale of the MAIA; MAIA-EA: Emotional Awareness subscale of the MAIA; MAIA-Trusting: Trusting subscale of the MAIA; VSI: Visceral Sensitivity Index; CAQ: Cardiac Anxiety Questionnaire; SBC-D: Dissociation subscale of the SBC.

### Outcomes of interest

Self-reported interoception measures (SIMs) were extracted from the studies, including each dimensional subscale when possible (e.g., the eight MAIA subscales). We also extracted psychological distress measures, encompassing stress, anxiety, depression, and PTSD symptoms (Supplementary Table S2) and mindfulness measures, encompassing total Five Facet Mindfulness Questionnaire (FFMQ) scores, Freiburg Mindfulness Inventory, etc. (Supplementary Table S3). We contacted authors to provide data for SIMs where necessary; for example, when manuscripts did not report scores for individual MAIA subscales. Come Studies were eligible for inclusion in main analysis if they reported outcomes on SIMs, even if they did not report mindfulness or distress measures.

### Systematic search

We conducted two searches on July 7th of 2024 of PubMed, ProQuest, PsycArticles, and WebOfScience based on title and abstract. Search One: (mindfulness OR meditation) AND (interocept* OR “bodily awareness” OR “body awareness” OR “somatic awareness” OR somatosensory OR visceral OR proprioception) AND (“questionnaire” or “inventory” or “scale” or “rating” or “instrument”). Search two: (mindfulness OR meditation) AND (BAQ OR MAIA OR SAQ OR PBCS OR BPQ OR VSI OR “Body Perception Questionnaire” OR “Body Awareness Questionnaire” OR “Self-Awareness Questionnaire” OR “Multidimensional Assessment of Interoceptive Awareness” OR “Visceral Sensitivity Index”). We additionally searched reference lists of identified studies and conducted an AI-assisted search on Undermind which supplied articles matching a natural language query (Supplementary Text S1). Only English language studies published before July of 2024 were included.

### Study selection

Search results consisting of citation data and abstracts were first screened for duplicate publications. Next, all abstracts were evaluated based on two main criteria: an empirical study (examples of excluded articles were review papers and protocol papers) and content relevance (based on mindfulness and interoception or body awareness). Remaining studies were screened by reviewing the methods section and full paper to further evaluate whether the study met all inclusion criteria. All inclusion/exclusion coding was conducted in duplicate, once by IT, and then independently by CV or YY.

### Data collection process

A coding manual was developed by the first author to guide the extraction of study descriptive and effect size data. Extraction of these data were conducted by IT and confirmed independently by CV, CW, YY, or JQ. Coding disagreements were discussed by the team.

### Data items

We extracted the following descriptive variables: duration of intervention in training weeks, subjects in mindfulness and control conditions (after dropout), mindfulness condition type, control condition type, age, percentage female (using reported gender), population type (normative, physical health condition, psychopathology, and other), and interoception measure/dimension. Intervention type was determined as following: (1) ‘mindfulness-based programs’ (MBPs) involved manualized interventions of at least 8 weeks that closely followed MBSR or MBCT, (2) ‘body-based interventions’ involved meditation but also extensive body-focused practices e.g. massage, sexual awareness, eating awareness and hunger monitoring, and (3) ‘other MBIs’ interventions included the small set of heterogeneous studies remaining. Interoceptive awareness dimensions were defined and categorized by expert input from SK and WM in the context of a maladaptive/adaptive framework from^37,49^ where interoception consists of sensing, attending, interpreting, and may be maladaptive or adaptive (maladaptive is not continuous with adaptive). The categorizations of study measures are shown in Table 1.

In the case of one study with two similar control groups (both active), we combined the groups (and pooled their means and standard deviations)^50^. Intervention impacts on interoception were measured as standardized mean differences, corrected using Hedges’ *g* adjustment for small samples^51^, with positive effect sizes favoring the mindfulness group over the control group. For calculating standardized treatment effects, we applied the following approaches (in decreasing preference): (1) Group differences adjusted for baseline measures (using analysis of covariance or regression adjustment) (2) Group differences in provided group means of change scores (3) Group differences in calculated group means of change scores (e.g., post mean intervention - pre mean intervention). Other reported statistics (e.g., mixed effect model estimates) were used when appropriate based on standard meta-analytic methods^52,53^.

### Risk of bias

Bias assessment was adapted from the ROB-2^54^, with modifications to fit the focus of the current study. As the study exclusively involved self-report measures, evaluation of bias related to objective outcome measurement was deemed not applicable. The bias evaluation process was as follows: At least two authors of IT, YY, CW, JQ and CV independently rated the risk across several domains, including (1) randomization bias, (2) bias due to deviations from intended interventions (e.g., issues related to blinding in the present study), (3) bias due to missing outcome (i.e., objective measurements of attrition) (4) outcome bias (i.e., attrition differences between groups) and (5) reporting bias. This estimated risk in each domain was then compared between raters. Any disagreements were discussed and resolved by a third author, YY. After inter-rater agreement was reached (Cohen’s *κ* = 0.71), studies were classified as having low, some concerns, or high risk of bias. Methods for quantifying study quality are detailed in supplement (Supplementary Text S2).

### Analysis

Analysis was conducted in R 4.3.1 using the *metafor* and *MAd* packages^55,56^. For RQ1, to assess overall mindfulness intervention effects on interoception, we conducted a correlated-and-hierarchical effects (CHE) meta-analysis with robust variance estimation, fitted using REML^41^. This model allows for inclusion of effect size estimates for multiple outcomes from the same study (e.g., subscale of a single interoception measure or multiple measures of interoception) and makes tentative assumptions about the intercorrelation between such estimates. We developed these assumptions using correlations between the outcomes reported in the literature in order to specify an assumed sampling variance-covariance matrix (Supplementary Table S4). We used robust variance estimation methods to allow for the possibility that these and other model assumptions about the variability of the effect size estimates might not be correct. We reported the overall average effect size estimate, heterogeneity estimates (tau) at the study level and effect size level, and a 67% prediction interval describing the distribution of effects that would be observed in new studies. We conducted sensitivity analysis by repeating the summary meta-analysis while omitting one primary study at a time, and by winsorizing outlier effect sizes.

We then (RQ2) examined for whom and under what circumstances mindfulness interventions produce effects on interoception measures i.e. testing meta-analytic moderators. Using separate meta-regression analyses for each moderator, we examined sex, age, mental health condition vs. normative, physical health condition vs. normative, sample size, duration of intervention (weeks and minutes, with and without home practice), preregistration status, control type (passive vs. active), control type (includes mind-body characteristics or not), adherence (session attendance) and quality of study (ROB-2). Meta-regression models were estimated using the same working model as in RQ1. To compare meta-analytic effects across intervention types with two levels, we used the clubSandwich package to conduct cluster-robust *t* tests with Satterthwaite degrees of freedom. To compare meta-analytic effects across intervention types or self-report measures with more than two categories, we used the *wildmeta* package^57^ to conduct Wald tests of the null hypothesis that average effects are equal across categories, with clustered wild bootstrapping to determine significance levels of the tests. While we calculated meta-analytic effects for all measures, we did not test for differences among all self-report measures statistically, given the large number of measures. Instead, we tested for differential effects between adaptive and maladaptive dimensions of interoception, provided that at least four studies included measures for a given dimension. We also statistically compared MAIA vs. other self-report measures, given the preponderance of studies using the MAIA.

To examine (RQ3) the relationships between interoception, distress and mindfulness, we assessed relationships between the pre-post effects in each domain. We provide scatterplots as well as Pearson’s and Spearman’s correlation coefficients where appropriate so as to provide information about the size of relationships. The observed correlation coefficients imply both baseline and latent change contributions to the effect and thus were not assessed for statistical significance. We were unable to disentangle the contributions using bivariate meta-analytic models due to a limited number of studies (see Deviations). On an exploratory basis we examined whether effects on mindfulness were significantly different from effects on interoception.

### Selective reporting and publication bias

Psychological research is affected by selective reporting bias (occurring when affirmative and statistically significant results have a higher probability of getting published^58^ and so-called ‘data contingent’ analyses^59^). We used different approaches to evaluate potential bias due to preferential reporting of statistically significant results. We deployed funnel plots to detect if small samples disproportionately report larger effects as well as a cluster-robust Egger’s regression test to test for significance. In addition, we evaluated three-parameter selection models (using the *metaselection* package) which explicitly model the probability that a finding is reported based on the sign and statistical significance level of the effect estimate. To account for correlations between effect sizes, we used cluster-wise bootstrapping to calculate percentile confidence intervals for the selection model parameters.

## Results

### Selected studies

**Fig. 1:**
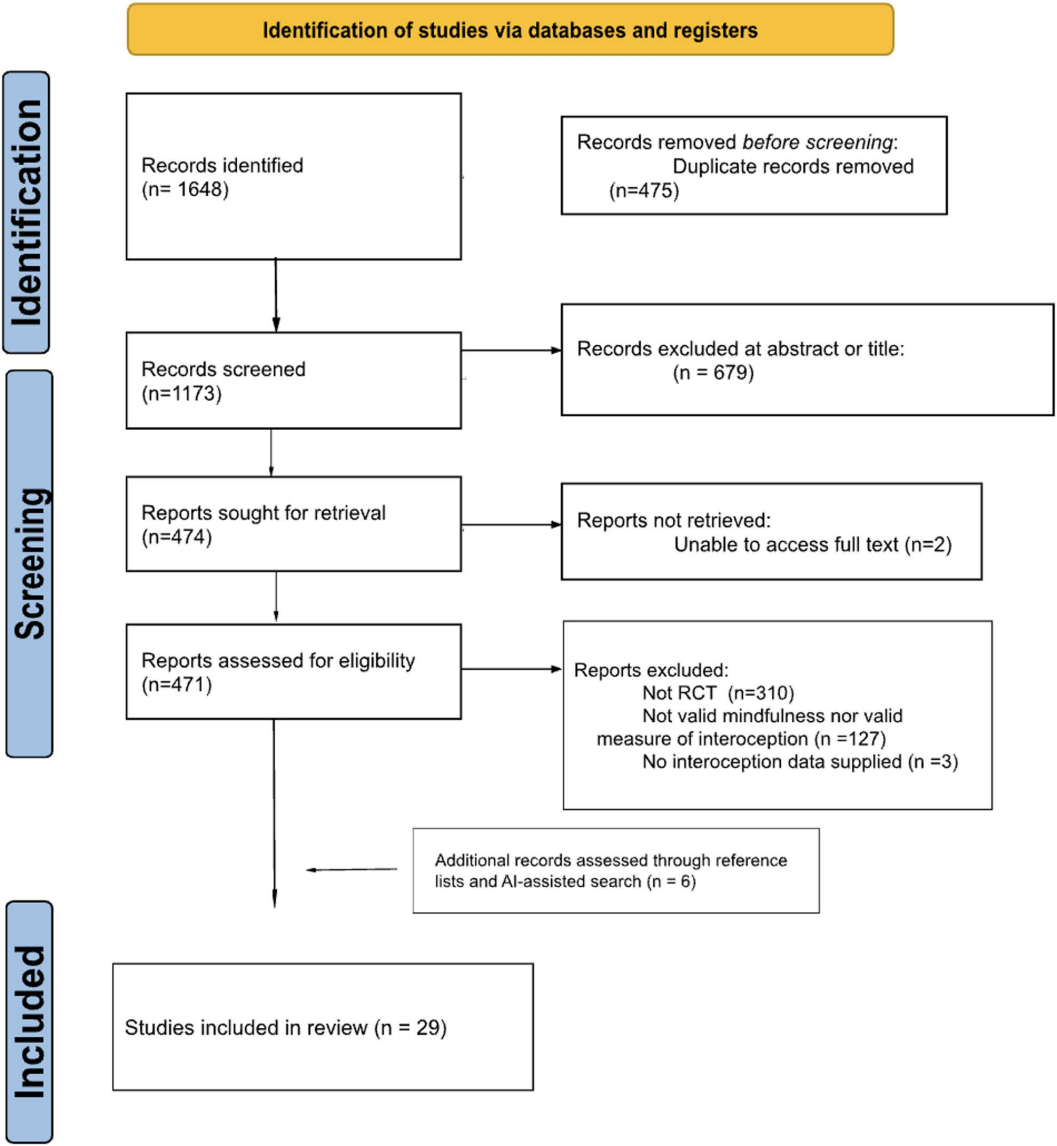
PRISMA^42^ flow diagram depicting number of identified and evaluated articles.

A PRISMA flow diagram is shown in Fig. 1.

### Study characteristics

**Fig. 2:**
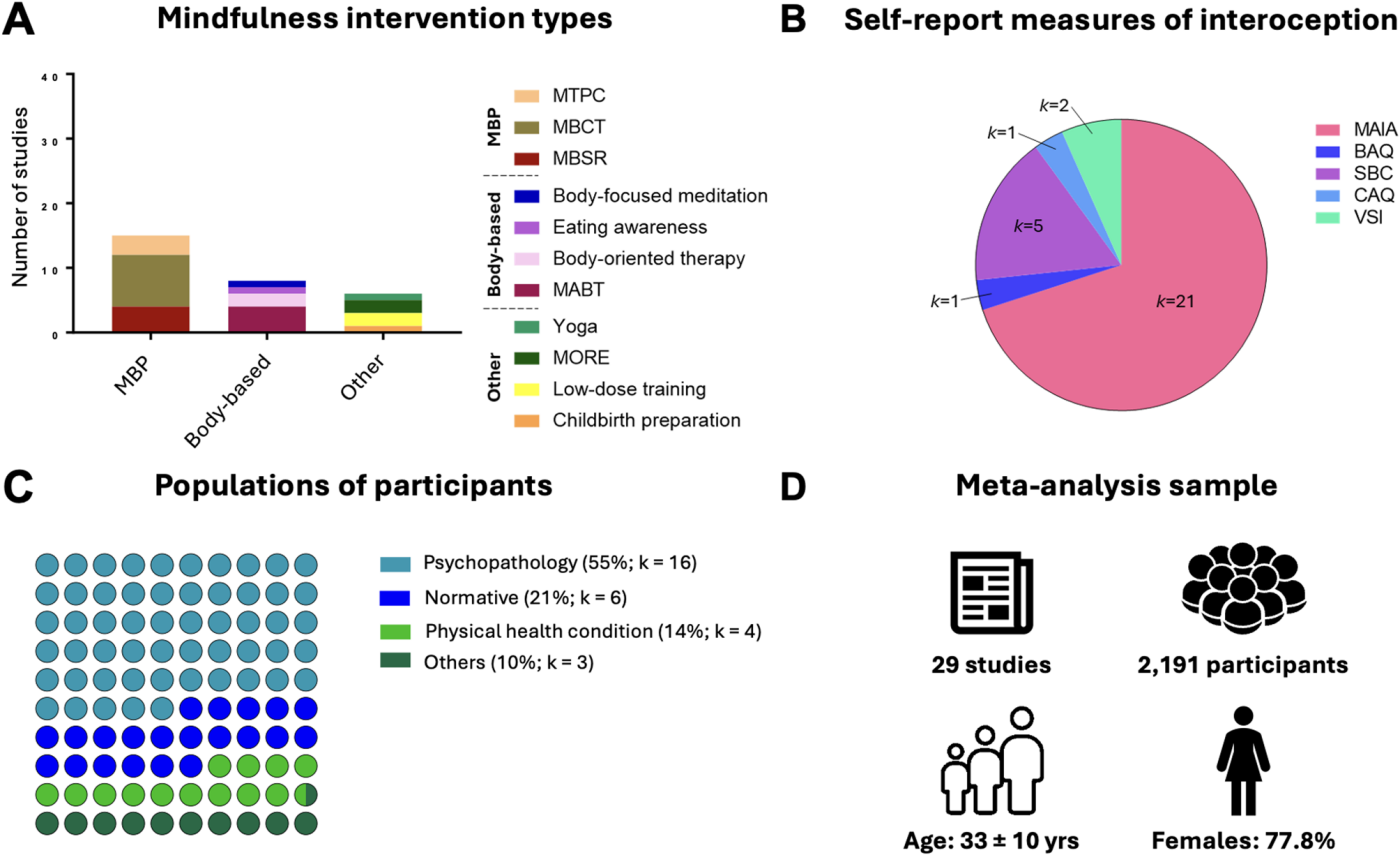
Summary of study characteristics included in the meta-analysis. (**A**) Types of mindfulness interventions categorized as mindfulness-based programs (MBP), body-based interventions, and other mindfulness interventions. Stacked bars indicate specific intervention subtypes. (**B**) Self-report measures of interoception used across studies, with the number of studies (*k*) per measure. *Note: Some studies included multiple self-report measures.* (**C**) Participant populations classified as having psychopathology, physical health conditions, normative status, or other. Percentages reflect the proportion of studies in each category relative to the total number of included studies, with the number of studies (*k*) shown for each group. (**D**) Characteristics of the overall sample included in the meta-analysis. MBSR: Mindfulness-based stress reduction; MBCT: Mindfulness-based cognitive therapy; MTPC: Mindfulness training for primary care (now called Mindful Behavior Change); MABT: Mindful awareness in body-oriented therapy; MORE: Mindfulness-oriented recovery enhancement; MAIA: Multidimensional Assessment of Interoceptive Awareness; BAQ: Body Awareness Questionnaire; SBC: Scale of Body Connection; CAQ: Cardiac Anxiety Questionnaire; VSI: Visceral Sensitivity Index.

Twenty-nine randomized controlled mindfulness interventions with self-report interoception measures were identified. For an overview of study characteristics, see Fig. 2. The studies involved 2,191 participants (*M* = 32.78 yrs, SD = 10.33 yrs), the majority of whom were female (77.82%). No studies involved children or adolescents, indicating a possible lack of investigation of mindfulness-based interoception changes in developmental populations. Sixteen studies involved participants with mental health conditions (trauma, depression, substance use, chronic pain of non-physical origin), six involved normative samples, four involved physical health conditions, and the remaining three had other characteristics (e.g. pregnancy;^60^). The average sample size for the mindfulness interventions was 44.4 participants, and 36.8 participants for the control interventions.

The majority of mindfulness-based interventions were mindfulness-based programs (e.g., MBSR), encompassing 15 studies (*n =* 1,255 participants). Thirteen of these studies were of the standard length (8 weeks) with one of 9 weeks^61^ and one of 12 weeks^62^. Three of the studies used identical programs, control conditions, and measures with different samples, and the authors provided data in aggregate^63–65^. These studies were collapsed for synthesis. The second most common category was body-based mindfulness (*k* = 8, *n* = 581), including studies that involved mindfulness meditation supplemented with massage (*k* = 5, e.g^66^). The majority of these studies were 8 weeks, with two more extended training programs^67,68^. Finally, six remaining studies (‘other’) (*n* = 426) involved mindfulness practices but did not follow a standardized program; these were variable in length (1–9 weeks).

Eighteen of the twenty-nine studies had active controls (e.g. relaxation, low-dose comparators, therapy, massage without meditation). Sixteen studies were preregistered, although interoception was only a primary outcome in three studies^50,68,69^ (psychological distress was more typical). Measures of interoception mainly consisted of the MAIA (21/29) followed by the SBC-Awareness (5/29), SBC-Dissociation (3/29), CAQ (1/29), BAQ (1/29) (see Table 1 for further categorizations).

### Risk of bias

**Fig. 3:**
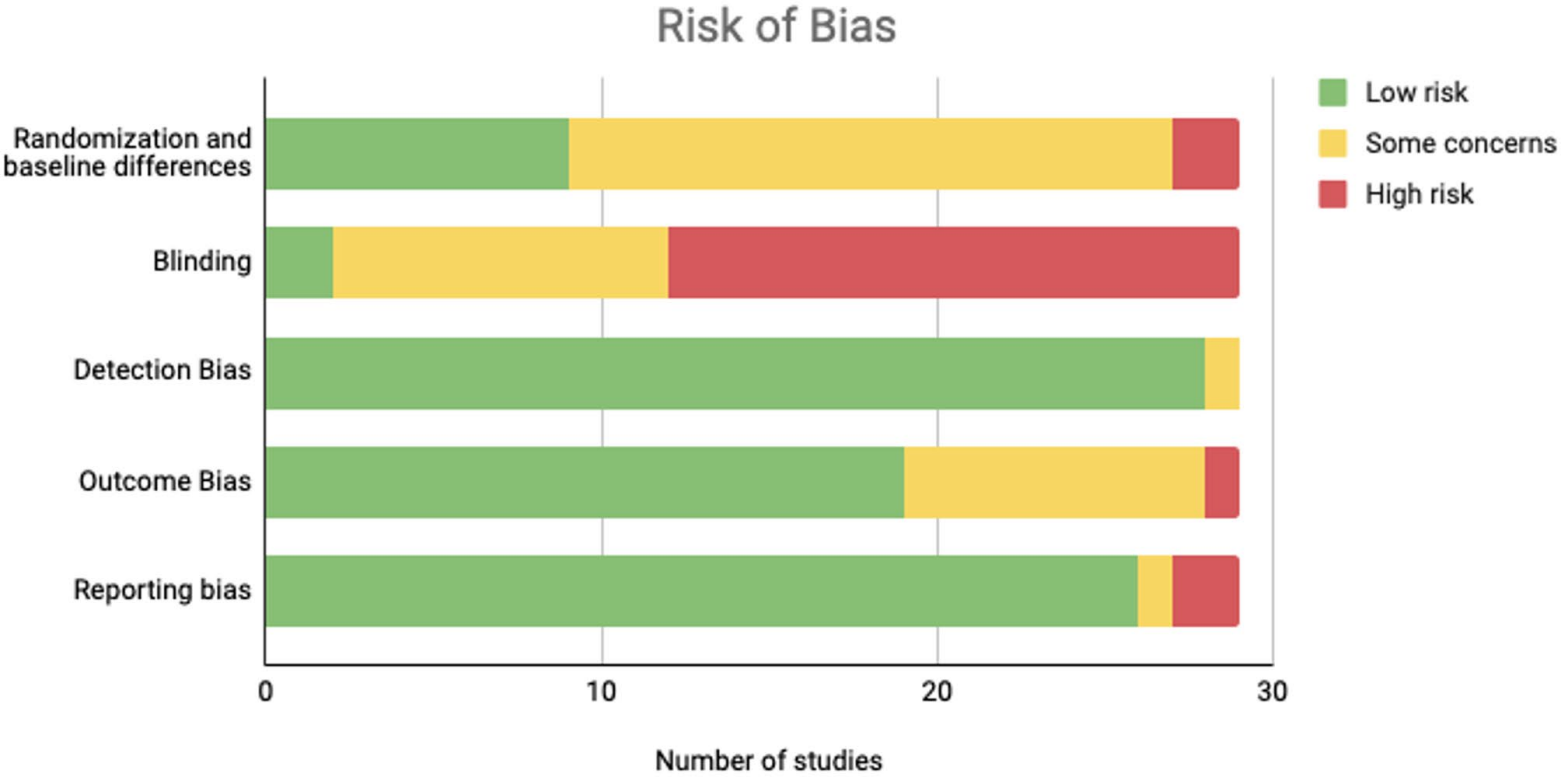
Study risk of bias by domain (ROB-2). “Low risk” categorization occurred when a study was judged to be free from bias in that domain; all criteria were met and deviations were unlikely to affect the outcome. “Some concerns” categorization occurred when a study was judged to have potential for bias in that domain; one or more criteria were not clearly met, raising uncertainty about the risk of bias. “High risk” categorization occurred when a study had clear evidence of bias or contained multiple issues that substantially lowered confidence in the results for that domain.

Risk of bias is shown in Fig. 3 and Supplementary Figure S1. The primary source of bias across studies was related to blinding (*k* = 17), as the majority of studies did not have instructors blinded to the intervention aims. Many studies were rated as ‘some concerns of bias’ for randomization, as many studies did not perform allocation concealment, and a few contained meaningful baseline differences between treatment and control groups. Most studies showed adequate reporting, and differential attrition between group was not a major area of potential bias. A full summary of study risk of bias is found in Supplementary Table S5.

### Study synthesis (RQ1)

**Fig. 4:**
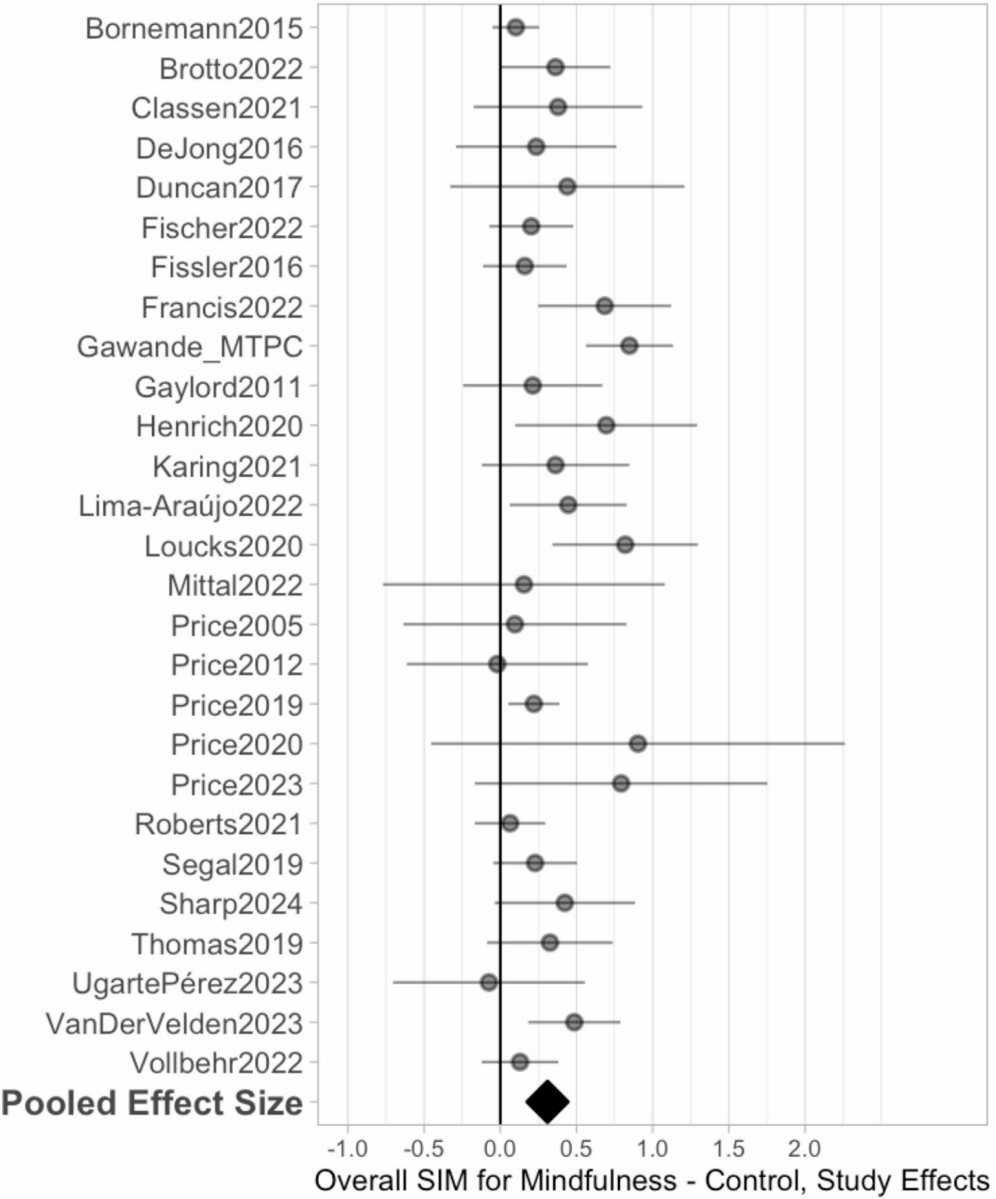
Forest plot showing change in self-reported interoception across all mindfulness-based interventions.

Across all studies, there was a positive, small-to-medium average effect on self-reported interoception (*k* = 29, 89 correlated effects, *g* = 0.31, *p* < 0.001, 95% CI = [0.21, 0.42]) (Fig. 4). Heterogeneity was low-to-moderate, with a total heterogeneity $\:\widehat{\tau\:}$ of 0.16 (between-study $\:\widehat{\tau\:}$ = 0.14, within-study $\:\widehat{\tau\:}$ = 0.09) and a 67% prediction interval of [0.15, 0.48]. We found no evidence of publication bias either through funnel plots (Supplementary Figure S2), Egger’s robust regression test (*p* = 0.41), or three-parameter selection models. Counter to typically expected selective reporting processes, the three-parameter selection model estimates indicated selection *in favor* of non-affirmative findings ($\:{\lambda\:}_{1}$ = 1.19, CI = [0.46, 2.99]), leading to a slightly larger adjusted average effect size estimate of *g* = 0.35, CI = [0.23, 0.51].

The omnibus effect was robust to outliers; the maximum change in average effect size based on leave-one-study-out sensitivity was +/-15%, and winsorizing did not change the average effect size estimate.

### Moderation analyses (RQ2)

**Fig. 5:**
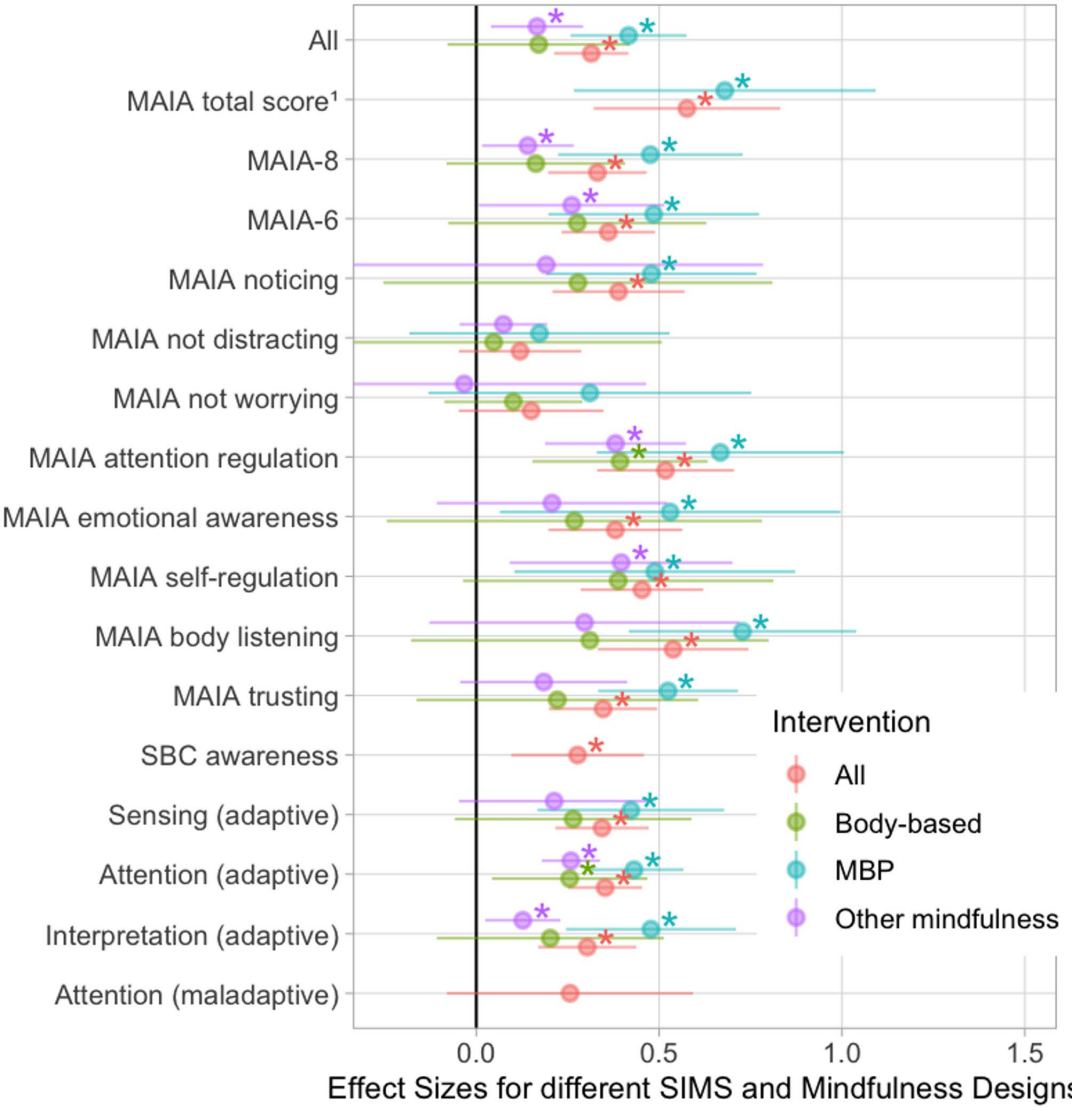
Forest plot depicting the effect of mindfulness intervention by intervention type and SIM dimension. Measures reported in more than four independent studies are shown. MAIA: Multidimensional Assessment of Interoceptive Awareness. ^1^MAIA total score, refers to reporting of effects on total score only (no aggregation of subscales); MAIA8: includes total score and aggregation of all eight MAIA subscales; MAIA6: does not include total score, but is aggregation of all MAIA subscales but noticing and not-worrying. SBC: Scale of Body Connection; * *p* < 0.05.

All effect sizes are shown for intervention type (design) and measures that include more than four independent studies in Fig. 5. We statistically examined design and interoception measures independently to maintain adequate power for moderation comparisons. Effects for all designs were significant: MBPs (*k* = 15, *g* = 0.41, *p* < 0.001, CI = [0.29,0.54]), body-based interventions (*k* = 8, *g* = 0.19, *p* < 0.05, CI = [0.032, 0.35]), and other mindfulness (*k* = 6, *g* = 0.23, *p* < 0.05, CI = [0.053,0.40]). There was no overall significant moderation by design (*p* = 0.14), but pairwise contrasts revealed MBPs had larger effect sizes than body-based interventions (B = 0.22, *p* = 0.048). For self-report measures, we compared the constructs of adaptive sensing (*g* = 0.29, *p* < 0.001, CI=[0.16,0.42]), adaptive interpretation (*g* = 0.28, *p* < 0.001, CI=[0.19, 0.38]), adaptive attention (*g* = 0.31, *p* < 0.001, CI=[0.20,0.42]), and maladaptive attention (*g* = 0.25, *p =* 0.1, CI = [-0.054,0.55]). The four-way contrast was not significant (*p* = 0.83), and no pairwise contrasts were significant (*p* > 0.4). Comparisons between SIMs were similar when examining MBP studies only. Of the SIMs, MAIA not-worrying (*g* = 0.15, *p* = 0.11, CI= [-0.048,0.35]) and MAIA not-distracting (*g* = 0.12, *p* = 0.11, CI= [-0.048,0.29]) showed the smallest effect sizes of any measure (Fig. 5).

No other moderators (e.g. active vs. passive controls) were statistically significant (unadjusted *p*s > 0.2) (Table 2). When examining only MBPs, likewise, no significant moderations were observed (Supplementary Table S6).

**Table 2:** Moderation effects across all studies.

Name	Coding	Beta	CI-Low	CI-High	*P*-value
Percent female	Continuous	0.00	− 0.007	0.004	0.523
Mean age	Continuous	− 0.01	− 0.019	0.005	0.225
MH sample vs. not	Binary	− 0.06	− 0.269	0.156	0.581
MH sample vs. normative	Binary	− 0.09	− 0.523	0.350	0.629
PH sample vs. not	Binary	− 0.02	− 0.405	0.366	0.887
PH sample vs. normative	Binary	− 0.04	− 0.466	0.382	0.809
Sample size	Continuous	0.00	− 0.003	0.004	0.724
Weeks intervention	Continuous	− 0.01	− 0.039	0.017	0.358
Quality	Continuous	0.01	− 0.062	0.078	0.784
Practice min excluding home	Continuous	− 0.04	− 0.249	0.163	0.378
Practice min including home	Continuous	− 0.01	− 0.386	0.366	0.913
Session attendance	Continuous	0.67	− 2.045	3.377	0.545
Preregistered	Binary	0.02	− 0.198	0.244	0.826
Active control	Binary	0.11	− 0.134	0.346	0.355
Mind-body control	Binary	0.14	− 0.446	0.724	0.525

Betas represent standardized effect for relationship between effect sizes and moderator. No FDR-correction was applied as no *p*-values met significance. CI: confidence interval; MH Sample: mental health condition sample; PH Sample physical health condition sample.

### Bivariate analyses (RQ3)

**Fig. 6:**
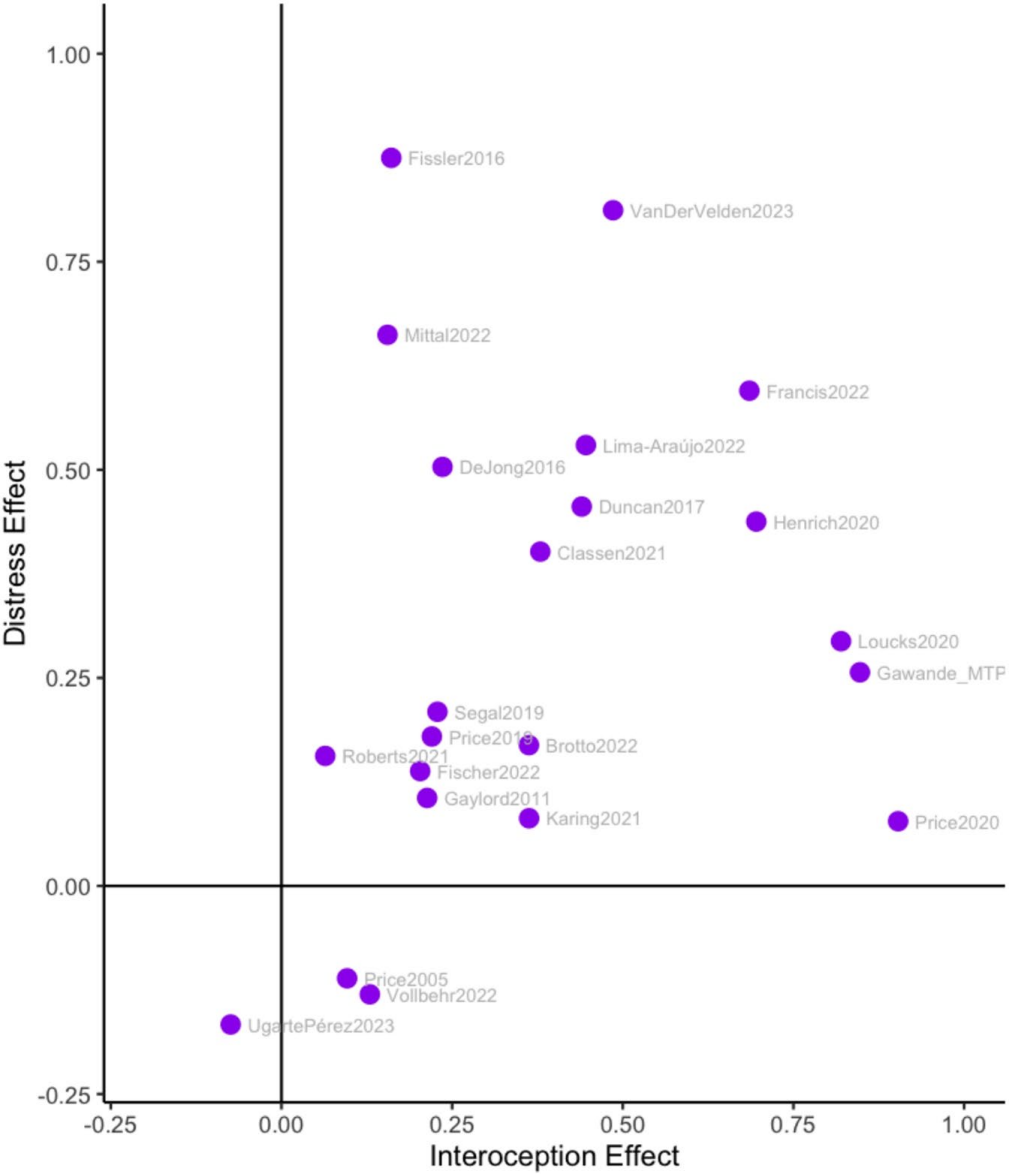
Changes in interoception vs. changes in distress. Each dot represents a study level effect, where positives represent improvements in interoception and decreases in distress.

We examined changes in distress and mindfulness for the studies that reported those outcomes. Effect sizes on distress were small-to-moderate (*k* = 24, *g* = 0.28, *p* < 0.001, CI = [0.16, 0.39]). Effect sizes on mindfulness were small-to-moderate (*k* = 17, *g* = 0.27, *p* < 0.001, CI = [0.16,0.38]). In the sample of 17 studies reporting mindfulness, the effect sizes on self-reported interoception was slightly larger (*g* = 0.34), although the difference was not statistically significant (*p* = 0.1). The relationship between increases in self-reported interoception and decreases in distress was positive (*r*(22) = 0.25, *rho*(22) = 0.36) (Fig. 6). The relationship between distress and mindfulness (*r*(14) = 0.77) was strong, and the relationship between mindfulness and self-reported interoception (*r*(13) = 0.36) was positive(Supplementary Figure S3 and S4). Bivariate model estimates were unstable and highly sensitive to sampling covariance assumptions, likely because the quantity of studies reporting multiple types of outcomes was too small (e.g., *k* < 50) to estimate correlations between the latent variables of impacts on distress and impacts on mindfulness. Consequently, we did not interpret the parameter estimates from the bivariate model.

## Discussion

In the current meta-analysis, we conducted the first systematic evaluation of self-reported interoception in studies employing mindfulness-based interventions. Across 29 studies with over 2,000 participants, we identified a small-to-moderate positive effect (Hedge’s *g* = 0.31) across all designs and measures. Prediction intervals did not cross zero, suggesting that future mindfulness interventions are likely to yield beneficial self-reported interoception outcomes with reasonable certainty. Heterogeneity was low-to-moderate, and publication bias based on statistical significance was not evident. In terms of moderation, Mindfulness Based Programs, based on MBSR, had larger effects than the body-based approaches included in our analysis. Lastly, we identified positive relationships between interoception improvements and decreases in distress, although we were unable to assess statistical significance based on the limited number of studies available.

First, we discuss the size and implications of the omnibus effect. Prior studies of objective body awareness measures found small effects with wide confidence intervals (e.g., *r* = 0.13, CI = [-0.36 to 0.62]^32^), which were limited to certain types of study designs and measures. The current result focusing on SIMs was larger, more precise, and consistent across study designs and measures. Other researchers have acknowledged that mindfulness and other behavioral interventions may change self-report more than objective measures^45,70^, but until the current study, there was little meta-analytic evidence to support these statements. The observed effect on SIMs was in the range of effects observed on trait mindfulness (Hedge’s *g*s = 0.28–0.49^46^), which is often used as a primary measure of treatment efficacy in mindfulness interventions. Notably, in studies from our sample that included both mindfulness and interoception outcome measures, the effects on interoception tended to be larger. Given that the therapeutic approach of programs like MBSR explicitly cultivates the process of sensing and interpretation of body signals^21^, our findings provide support for the use of SIMs as primary measures in mindfulness studies.

Several studies included here have reported the role of interoceptive awareness measures or interoceptive dimensions as mediators or partial mediators of clinical outcomes in MBIs, especially in populations with depression. For example, Fissler et al.^71^ reported that the effects of a brief mindfulness intervention on depression symptoms were mediated by changes in a path where score increases on multiple MAIA subscales were serially related to decentering, which reduced depressive symptoms. DeJong et al.^72^ reported that among a population of patients with depression and chronic pain, the positive effect of MBCT on depression severity was mediated by MAIA, specifically the ‘not distracting’ subscale. Finally, Schuman-Olivier et al.^38^ found that the effect on health behavior change initiation from a MBP among primary care patients with depression was partially mediated by MAIA ‘Body Trusting’, while health behavior change was mediated by ‘body listening’ among non-depressed comparators. These studies suggest that certain self-report measures of interoceptive awareness may be clinically relevant mediators. However, the most optimal shifts in interoceptive processing may differentially depend on disorder and outcome measured. As we only compared general categories of psychopathology, physical health conditions, and normative populations in the current meta-analysis, these differences need further study.

Our meta-analytic findings support the concept that interoception, and particularly the self-reported aspect of interoception, is a key process targeted in mindfulness training. Models of mindfulness and interoception tend to emphasize formal meditation practice, pointing to how practices like body scans and breath attention could gradually change one’s way of attending to internal sensations^73^. Repeated practice may cultivate the habit of attending to sensations without overt emphasis on regulating, avoiding, or suppressing them, leading to positive downstream effects on emotional experience^17,74^. However, numerous questions remain about the potential underlying mechanism(s) of action. For one, there was no effect of study quality, attendance or duration. This could be viewed as evidence of a general interoceptive appreciation or attentional effect, instead of an effect specific to the involved meditation techniques. Prior studies have found inconsistent relationships between the ‘dosage’ of formal practice (usually measured in terms of hours or practice) and effects on distress^75^. Thus it is possible that the quantity of practice hours is less informative than the depth of learning from the intervention, for which phenomenological interviewing may be more informative^76^.

Another noteworthy finding was that MBPs were associated with greater improvements in interoception than body-based studies, although both were significant. Body-based MBI studies included here involved components like massage in the therapeutic process, where touch supports a non-judgemental awareness of the body. Many of these studies used the Mindful Awareness in Body-Oriented Therapy (MABT) protocol, an individual therapy that integrates touch, mindfulness, and psychoeducation to foster interoceptive awareness for self-care by increasing awareness of sensations and emotions and improving somatic appraisal^19^. A nuance of these studies is that many of the control conditions also incorporated massage, which may have decreased the size of treatment effects on interoception due to the common anchoring in modulation of body states^77^. However, when we coded control conditions based on mind-body components, this did not moderate overall treatment effects. More insight into this nuanced finding is necessary. Future studies could conduct dismantling approaches (e.g. mindfulness + massage, mindfulness, massage) or administer state measures after sessions to identify active components.

A primary motivation for our study was examining differential effects of MBIs on distinct interoception self-report measures. This potential was limited by the preponderance of studies employing the MAIA. The MAIA involves 8 subscales, which were designed to measure positive aspects of mind-body interventions on interoception^35^. Our construct-forward approach was to decompose the MAIA and other scales into adaptive and maladadaptive interoception, with sensing, attention, and interpretation components^37,49^. Many studies just report the total MAIA scores, but we contacted study authors and generated sufficient studies to measure effects in all positive categories (e.g. *k* = 16 for adaptive interpretation), and also in maladaptive attention (*k* = 6). However, we did not find any statistically significant evidence of differences across categories. An exploratory analysis revealed smaller effects on ‘not-distracting’ and ‘not-worrying’ subscales. However, this could simply reflect limited construct validity in the original MAIA subscales^36^. In contrast, moderation effects by objective body awareness measures have been identified (more distal, emotionally targeted measures show larger effects than interoceptive accuracy measures;^32^). Objective measures may provide the discriminability that is lacking in self-report measures.

One may still question whether interoceptive benefits of mindfulness are epiphenomenal to improvements in distress. A formal test of this possibility would require mediation analyses, and would thus be reliant on individual participant data. In the current meta-analysis, we primarily had access to study-level data and thus attempted to assess whether changes in interoception were associated with changes in distress. Our findings suggest the possibility of a positive relationship, where larger changes in interoception wer asssociated with larger decreases in distress. The relationship between mindfulness and distress appeared stronger, but baseline associations are also stronger in mindfulness^78^. Indeed, both apparent relationships could be due in whole or part to the associations between the measures (i.e., sampling errors;^41^) rather than due to relation between the latent impacts; our sample did not include a sufficient number of studies effectively isolate the relation between latent impacts (50 or more may be required^79^). Thus, one future question is whether changes in objective measures in interoception show a similar relationship to distress.

## Limitations

A limitation of the current literature is that it may not be advanced enough to meta-analytically identify differential effects of specific aspects of self-reported interoceptive awareness. As mentioned previously, the vast majority of studies used the MAIA. While the MAIA has different dimensions of interest, some of these reflect overlapping latent constructs, tend to be moderately to highly correlated, and share similar language. This may have limited our ability to disentangle key components of interoception like sensing, attention, and interpretation. The generalizability of the relationship between mindfulness and interoception may also be limited by the limited range of mindfulness trainings. For example, we did not include single-session or app-based interventions and study populations, and, there were no developmental samples available. Another limitation was that we were unable to meta-analytically examine mediation effects or other causal modelling findings, as too few studies conducted mediation. However, studies with larger sample sizes examining interoception as a mediator tended to find larger effects, suggesting that mediation is possible. Finally, we were unable to statistically examine relationships between interoception, distress, and mindfulness due to limited numbers of studies. Future examinations of this question would ideally draw on individual participant-level data from primary studies.

## Future directions

Very few of the selected studies examined both objective and subjective measures of interoception in parallel (we identified only one^69^. We propose that assessing both dimensions simultaneously is crucial to understanding whether and how mindfulness training alters the processing of interoceptive signals. The effects observed here on self-reported interoception appear to be more robust—i.e., larger—than those reported for objective measures, in the studies available to date^30,32,33^. This discrepancy may suggest that mindfulness primarily influences attitudes toward bodily sensations and regulatory aspects of interoception, rather than perceptual accuracy or acuity per se. Future studies should incorporate direct comparisons of objective and subjective interoceptive measures within the same samples to more clearly elucidate these mechanisms. Such work would likely need to focus on specific interoceptive channels, such as respiratory signals, which are particularly emphasized in many MBPs.

We also did not observe moderation effects based on mental or physical health conditions. This is consistent with prior individual participant data meta-analyses of mindfulness interventions that similarly failed to identify moderation by distress levels^79^. One possible explanation is the heterogeneity of clinical conditions studied across the included trials. Emerging small-sample studies do suggest that mindfulness may differentially affect interoceptive processing in depression versus anxiety^80^, although our analysis did not identify enough studies to formally test this possibility.

A striking gap revealed by our meta-analysis is the absence of studies examining the effects of mindfulness on interoception in youth and adolescent populations. Adolescence is a particularly sensitive developmental period marked by rapid changes in both interoception and the emergence of many mental health conditions^80–84^. Investigating whether and how mindfulness training influences subjective and objective interoception in adolescents could clarify mechanisms of resilience and inform early intervention strategies.

## Conclusion

This study represents the first meta-analysis of the effects of mindfulness-based interventions on self-reported interoception. We observed robust improvements across the 29 studies included, providing evidence supporting the idea that changes in interoception, or at least the self-reported aspects of it, may underpin the therapeutic effects of mindfulness training. Nevertheless, the lack of sensitivity to many qualities of the included studies like dosage or self-report measure require further investigation.

## Supplementary Information

Below is the link to the electronic supplementary material.

Supplementary Material 1

Supplementary Material 2

## Data Availability

The datasets and code generated during the current study are available at: [https://osf.io/qzyh5/?view_only=c537e7d6de084f32a90eb12792175a2e] .

## Declarations

### AI statement

No AI was used in the preparation of the language of this manuscript. An AI search was used to supplement the systematic search, and AI was used to generate Fig. 2.
