# Correction: A meta-analysis of the effects of mindfulness meditation training on self-reported interoception

**DOI:** 10.1038/s41598-025-32949-0

**Published:** 2025-12-23

**Authors:** Isaac N. Treves, Ya-Yun Chen, Caitlyn L. Wilson, Charles Verdonk, Joanne Qina`au, James E. Pustejovsky, Simon B. Goldberg, Wolf Mehling, Zev Schuman-Olivier, Sahib S. Khalsa

**Affiliations:** 1 Department of Psychiatry, New York State Psychiatric Institute, Columbia University Irving Medical Center, New York, 10032 USA; 2 Psychology, Virginia Tech, Blacksburg, 24061 USA; 3 Department of Psychology, University of Colorado Denver, Denver, 80204 USA; 4 French Armed Forces Biomedical Research Institute, Brétigny-sur-Orge, France; 5 UMR VIFASOM, Université de Paris, Paris, France; 6 Laureate Institute for Brain Research, Tulsa, OK 74136 USA; 7 Osher Center for Integrative Health, University of California, San Francisco, 94115 USA; 8 Educational Psychology Department, University of Wisconsin-Madison, Madison, WI 53706 USA; 9 Counseling Psychology, University of Wisconsin-Madison, Madison, WI 53706 USA; 10 Center for Mindfulness and Compassion, Department of Psychiatry, Cambridge Health Alliance, Cambridge, MA 02139 USA; 11 Department of Psychiatry, Harvard Medical School, Cambridge, MA 02139 USA; 12 Department of Psychiatry and Biobehavioral Sciences, Semel Institute for Neuroscience and Human Behavior, David Geffen School of Medicine, University of California at Los Angeles, 760 Westwood Plaza, Los Angeles, CA 90024 USA

Correction to: *Scientific Reports* 10.1038/s41598-025-22661-4, published online 06 November 2025

The original version of this Article contained an error in the name of author Joanne Qina`au, which was incorrectly published as Joanne Qinàau.

The original Article has been corrected.

