# Supplementary material for: A meta-analysis of the effects of mindfulness meditation training on self-reported interoception
Source: Sci Rep. 2025 Nov 6;15:38889. doi: 10.1038/s41598-025-22661-4 (PMC12592345; doi:10.1038/s41598-025-22661-4)
Supplement: Supplementary file 2 — Supplementary Material 2 [file 41598_2025_22661_MOESM2_ESM.docx]

**Supplement 1: A Meta-analysis of the Effects of Mindfulness Meditation Training on Self-reported Interoception**

Isaac N. Treves^1*^, Ya-Yun Chen^2,+^, Caitlyn L. Wilson^3,+^, Charles Verdonk^4,5,6^, Joanne Qina`au^7^, James E. Pustejovsky^8^, Simon B. Goldberg^9^, Wolf Mehling^7^, Zev Schuman-Olivier^10,11^, Sahib S. Khalsa^12^

^1^New York State Psychiatric Institute, Columbia University Irving Medical Center, Department of Psychiatry, New York, 10032, USA

^2^Psychology, Virginia Tech, Blacksburg, 24061, USA

^3^University of Colorado Denver, Department of Psychology, Denver, 80204, USA

^4^ French Armed Forces Biomedical Research Institute, Brétigny-sur-Orge, France

^5^ UMR VIFASOM, Université de Paris, Paris, France

^6^ Laureate Institute for Brain Research, Tulsa, OK, USA 74136

^7^ Osher Center for Integrative Health, University of California, San Francisco, 94115, USA

^8^ Educational Psychology Department, University of Wisconsin-Madison, Madison, WI 53706, USA ^9^Counseling Psychology, University of Wisconsin-Madison, Madison, WI 53706, USA

^10^Center for Mindfulness and Compassion, Department of Psychiatry, Cambridge Health Alliance, Cambridge, MA, 02139, USA

^11^Department of Psychiatry, Harvard Medical School, Cambridge, MA, 02139, USA

^12^Department of Psychiatry and Biobehavioral Sciences, Semel Institute for Neuroscience and Human Behavior, David Geffen School of Medicine, University of California at Los Angeles, 760 Westwood Plaza, Los Angeles, CA 90024, United States

*Isaac.Treves@nyspi.columbia.edu

^+^these authors contributed equally to the work

**Table of Contents**

[Text S1. Additional Search Details. 3](#_Toc208738842)

[Text S2. Study quality assessment and risk of bias scoring. 3](#_Toc208738843)

[Text S3. Coding of moderators. 3](#_Toc208738844)

[Table S1. Summary of included studies. 5](#_Toc208738845)

[Table S2. Measures of psychological distress. 12](#_Toc208738846)

[Table S3. Measures of mindfulness. 13](#_Toc208738847)

[Table S4. Literature correlations between SIMs for correlated-and-hierarchical effects models. 14](#_Toc208738848)

[Table S5. Risk of bias ratings. 15](#_Toc208738849)

[Table S6. Moderation analyses for MBP interventions. 16](#_Toc208738850)

[Figure S1. Overall quality of studies. 17](#_Toc208738851)

[Figure S2: Funnel plot of all effect size estimates. 17](#_Toc208738852)

[Figure S3. Changes in mindfulness vs changes in distress. 18](#_Toc208738853)

[Figure S4. Changes in interoception vs changes in mindfulness. 19](#_Toc208738854)

[References 20](#_Toc208738855)

Text S1. Additional Search Details.

- To add to the comprehensiveness of our literature search, we used AI tools which deploy natural language processing to identify matching literature based on overarching search goals (not exact matches of strings). The Undermind AI search (<https://www.undermind.ai/home/>) was:
  - “I want to find randomized controlled trials that investigate mindfulness meditation interventions using validated self-report measures of body awareness or interoception, regardless of population or specific type of mindfulness intervention.”
- We did not conduct any screening calibration.
- To identify grey literature, we included preprints and theses (which are indexed in ProQuest). We also searched reference lists of included papers.

Text S2. Study quality assessment and risk of bias scoring.

Study quality was assessed using an adapted version of the Cochrane Risk of Bias 2 (ROB-2) tool (Sterne et al., 2019), following PRISMA 2020 guidelines. The tool evaluates bias across five domains. (1) For the domain of bias arising from the randomization process, reviewers considered whether the method of sequence generation was appropriate, whether allocation was adequately concealed from study investigators, and whether there was evidence of randomization problems, such as baseline differences across groups. (2) Bias due to deviations from intended interventions was assessed by evaluating whether participants and treaters were blinded, and whether any deviations from the assigned intervention were likely to have influenced the outcomes. (3) For bias due to missing outcome data, we considered whether attrition was clearly and objectively reported, and whether dropout rates and reasons were transparently described. (4) Outcome-related bias was examined by evaluating the extent of attrition, whether it differed systematically between groups, and whether there were concerns that data were not missing at random. (5) Finally, risk of reporting bias was assessed by determining whether all pre-specified outcomes were reported, comparing published outcomes with those listed in clinical trial registrations (e.g., ClinicalTrials.gov), preprints, or protocol papers. In the absence of pre-registrations, we evaluated whether the reported outcomes matched those described in the methods section.

Each domain was scored on a three-point scale, where 2 indicated low risk of bias, 1 indicated some concerns, and 0 indicated high risk of bias. Two reviewers independently rated each study, and discrepancies were resolved through discussion or, when necessary, adjudicated by a third reviewer (YY). Final scores were summed to yield an overall score ranging from 0 to 10 and this was used in moderation tests; studies were not further classified into levels of overall risk. Full risk of bias ratings are provided in Supplementary Table S5.

Text S3. Coding of moderators.

The following variables were coded to include as moderators in analyses: sex, age, mental health condition, physical health condition, sample size, duration of intervention, preregistration status, control condition type, adherence, and study quality. **Sex** was coded as percentage of female participants based on biological sex and **age** was coded as mean age in years. If reported individually by group, a weighted mean was calculated for sex and age, respectively (i.e., mean per group x sample size per group / total sample size). **Mental health condition** was coded “yes” if the study included a clinical sample with depression, anxiety, addiction, or chronic pain. **Physical health condition** was coded “yes” if the study included a clinical sample with a physical health condition such as cancer or cardiac arrhythmia. **Sample size** was coded as total number of participants across groups after dropout, unless imputation took place, in which case it was the total before dropout. **Duration of intervention** was coded as both weeks (i.e., number of weeks the training took place) and minutes (i.e., number of total minutes of practice). Two variables for duration of minutes were calculated, one with home practice and one without home practice minutes. **Preregistration status** was coded as “yes” if the study mentioned that it was preregistered or was a clinical trial. **Control type (active)** was coded as “yes” if the control group involved engagement in some form of intervention or activity (e.g., meditation). **Control type (mind-body)** was coded as “yes” if the control group involved mind-body education or training (e.g., yoga, tai chi, exercise, dance, body scans, breath meditation). **Adherence** was coded based on session attendance and was calculated as either the mean number of sessions attended divided by number of sessions of the intervention, or the proportion of individuals who completed at least 75% of sessions. **Study quality** was coded based on Cochrane v2.0 and may be found in **Text S2.**

Table S1. Summary of included studies.

| Author | Sample characteristics | Intervention Length (weeks) | SIM | Intervention Group | Group n | Mindfulness Intervention Category | Intervention Group Description |
| --- | --- | --- | --- | --- | --- | --- | --- |
| Bornemann et al., 2015 | *Normative:*  Healthy adults, 54.74% female, M_age_ = 41.47 | 13.5 | MAIA | Mindfulness | 148 | Body-based | Interoceptive body awareness-focused meditations from ReSource Project |
|  |  |  |  | Control | 80 | - | *Passive:* Retest control |
| Brotto et al., 2022 | *Other:*  Women with SIAD, 100% female, M_age_ = 38.56 | 8 | MAIA | Mindfulness | 61 | MBP | MBCT + psychoeducation |
|  |  |  |  | Control | 68 | - | *Active:* Group supportive sex education and therapy |
| Classen et al., 2021 | *Psychopathology:* Women with childhood trauma, 100% female, M_age_ = 43.51 | 20 | SBC-A & SBC-D | Mindfulness | 18 | Body-based | Body-oriented group therapy for complex trauma |
|  |  |  |  | Control | 18 | - | *Passive:* Waitlist |
| de Jong et al., 2016 | *Psychopathology:* Patients with chronic pain and comorbid depression, 71.97% female, M_age_ = 50.8 | 8 | MAIA | Mindfulness | 14 | MBP | MBCT + TAU |
|  |  |  |  | Control | 12 | - | *Passive:* Waitlist + TAU (i.e., regular visits with psychiatrist, psychotherapist, pain physician, and antidepressant and/or pain medications) |
| Duncan et al., 2017 | *Other:*  Pregnant women in 3^rd^ trimester, 100% female, M_age_ = NR | 1 | MAIA | Mindfulness | 15 | Other mindfulness | Mindfulness-based childbirth preparation course |
|  |  |  |  | Control | 13 | - | *Active:* Standard childbirth preparation course |
| Fischer et al., 2022 | *Normative:*  Individuals with elevated perceived stress and physical stress-related symptoms, 89.55% female, M_age_ = 47.03 | 12 | MAIA | Mindfulness | 34 | MBP | Mindfulness training^a^ |
|  |  |  |  | Control | 33 | - | *Active:* Iyengar yoga |
| Fissler et al., 2016 | *Psychopathology:* Patients with depression, 56.76% female, M_age_ = 42 | 3 | MAIA | Mindfulness | 36 | MBP | MBCT |
|  |  |  |  | Control | 32 | - | *Active:* Rest training |
| Francis et al., 2022 | *Psychopathology:* Primary care patients with mild to severe mental health disorders, 72.8% female, age range: 20-72 | 8 | MAIA | Mindfulness | 45 | MBP | Mindfulness-integrated cognitive behavior therapy (MiCBT)^b^ |
|  |  |  |  | Control | 45 | - | *Passive:* TAU (i.e., medication and/or psychological therapy) |
| Gawande, To, et al., 2019^c^ | *Psychopathology:* Primary care patients with DSM-V diagnosis, 65.4% female, M_age_ = 40.5 | 8 | MAIA | Mindfulness | 65 | MBP | Mindfulness training for primary care (MTPC) |
|  |  |  |  | Control | 33 | - | *Active:* Low-dose mindfulness comparator |
| Gawande, Pine, et al., 2019^c^ | *Psychopathology:* Primary care patients with DSM-V diagnosis, 69% female, M_age_ = 44 | 8 | MAIA | Mindfulness | 36 | MBP | Mindfulness training for primary care (MTPC) |
|  |  |  |  | Control | 20 | - | *Active:* Low-dose mindfulness comparator |
| Gawande et al., 2023^c^ | *Psychopathology:* Primary care patients with DSM-V diagnosis, 58.9% female, M_age_ = 36.6 | 8 | MAIA | Mindfulness | 49 | MBP | Mindfulness training for primary care (MTPC) |
|  |  |  |  | Control | 24 | - | *Active:* Low-dose mindfulness comparator |
| Gaylord et al., 2011 | *Physical health:* Female patients with IBS^d^, % female, M_age_ = 42.72 | 8 | VSI | Mindfulness | 36 | MBP | Mindfulness-based stress and pain management program^a^ |
|  |  |  |  | Control | 39 | - | *Active:* Social-support group |
| Henrich et al., 2020 | *Physical health:* Female patients with IBS^d^, 100% female, M_age_ = 35.53 | 6 | VSI | Mindfulness | 25 | MBP | MBCT-IBS^b^ |
|  |  |  |  | Control | 23 | - | *Passive:* Waitlist |
| Karing & Beelmann, 2021 | *Normative:* University students, 80.3% female, M_age_ = 22.68 | 6 | SBC-A | Mindfulness | 32 | Other mindfulness | Low-dose mindfulness training^a^ |
|  |  |  |  | Control | 23 | - | *Passive:* Waitlist |
| Lima-Araújo et al., 2022 | *Normative:*  Healthy graduate and undergraduate students, 50% female, M_age_ = 24.15 | 1 | MAIA | Mindfulness | 20 | Other mindfulness | Brief mindfulness training |
|  |  |  |  | Control | 20 | 20 | *Active:* Psychoeducation |
| Loucks et al., 2020 | *Normative:* Undergraduate students, 68% female, M_age_ = 20 | 9 | MAIA | Mindfulness | 37 | MBP | Mindfulness-Based College^a^ |
|  |  |  |  | Control | 40 | - | *Active:* Waitlist + enhanced TAU (i.e., referral to study psychiatrist and university counseling resources) |
| Mittal et al., 2022 | *Psychopathology:* Patients with non-cardiac chest pain, 68% female, M_age_ = 54.2 | 8 | CAQ | Mindfulness | 18 | MBP | MBCT + TAU |
|  |  |  |  | Control | 6 | - | *Passive:* Waitlist + TAU (i.e., continuation of any general practitioner-prescribed treatment) |
| Ugarte Pérez et al., 2023 | *Physical health:* Individuals with BMI>24.9 and with dysregulated eating^d^, 76% female, M_age_ = 34.8 | 8 | MAIA | Mindfulness | 21 | Body-based | Mindfulness-based eating awareness training (MB-EAT) |
|  |  |  |  | Control | 20 | - | *Active:* Behavioral weight loss counseling |
| Price, 2005 | *Psychopathology:* Adult females in psychotherapy for child sexual abuse, 100% female, M_age_ = 41 | 8 | SBC-A & SBC-D | Mindfulness | 11 | Body-based | Body-oriented therapy |
|  |  |  |  | Control | 11 | - | *Active:* Massage therapy |
| Price et al., 2012 | *Psychopathology:* Women in substance use disorder treatment, 100% female, median age = 39 | 8 | SBC-A & SBC-D | Mindfulness | 24 | Body-based | Mindful awareness in body-oriented therapy (MABT) + TAU |
|  |  |  |  | Control | 11 | - | *Active:* Enhanced TAU (i.e., inpatient and individualized outpatient programs with psychoeducation, cognitive-behavioral therapy, and continuing care) |
| Price et al., 2019 | *Psychopathology:* Women in intensive outpatient program treatment for substance use disorder, 100% female, median age = 35 | 8 | MAIA | Mindfulness | 74 | Body-based | Mindful awareness in body-oriented therapy (MABT) + TAU |
|  |  |  |  | Control | 113 | - | *Active:* Women’s health education + enhanced TAU (i.e., intensive outpatient program with group sessions and individual counseling with psychoeducation focus) |
| Price et al., 2020 | *Psychopathology:* Individuals prescribed buprenorphine for opioid use disorder, 30% female, M_age_ = 46.6 | 8 | MAIA | Mindfulness | 5 | Body-based | Mindful awareness in body-oriented therapy (MABT) + TAU |
|  |  |  |  | Control | 5 | - | *Passive:* TAU (i.e., medication continuation + optional addiction counseling, mental health care, and medical care) |
| Price et al., 2023 | *Normative:*  Healthy individuals with self-reported elevated stress, 50% female, M_age_ = 36.1 | 8 | MAIA | Mindfulness | 11 | Body-based | Mindful awareness in body-oriented therapy (MABT) |
|  |  |  |  | Control | 11 | - | *Passive:* No treatment |
| Roberts et al., 2021 | *Psychopathology:* Patients with opioid-treated chronic pain, 66.32% female, M_age_ = 56.71 | 8 | MAIA | Mindfulness | 50 | Other mindfulness | Mindfulness-oriented recovery enhancement (MORE) |
|  |  |  |  | Control | 45 | - | *Active:* Support group psychotherapy |
| Segal et al., 2019 | *Psychopathology:* Outpatients with remitted depression, 67.46% female, M_age_ = 40.63 | 8 | BAQ | Mindfulness | 60 | MBP | MBCT |
|  |  |  |  | Control | 60 | - | *Active:* Group cognitive therapy |
| Sharp et al., 2024 | *Other:*  Pregnant people at risk for hypertension, 100% female, M_age_ = 32.52 | 8 | MAIA | Mindfulness | 12 | MBP | Phone-delivered mindfulness training^a^ + TAU |
|  |  |  |  | Control | 12 | - | *Passive:* TAU (i.e., usual obstetric care and weekly check-ins) |
| Thomas et al., 2019 | *Physical health:* Overweight and obese female cancer survivors^d^, 100% female, M_age_ = 57.92 | 10 | MAIA | Mindfulness | 15 | Other mindfulness | Mindfulness-oriented recovery enhancement (MORE) + exercise and nutrition counseling |
|  |  |  |  | Control | 15 | - | *Active:* Exercise and nutrition counseling |
| van der Velden et al., 2023 | *Psychopathology:* Patients with recurrent depression, 74.42% female, M_age_ = 43.93 | 8 | MAIA | Mindfulness | 42 | MBP | MBCT + TAU |
|  |  |  |  | Control | 24 | - | *Passive:* TAU (i.e., medication support only – stable dose of antidepressant or no medication) |
| Vollbehr et al., 2022 | *Psychopathology:* Young women with depression 100% female, M_age_ = 25.05 | 9 | SBC-A & MAIA-TR | Mindfulness | 86 | Other mindfulness | Mindful yoga intervention + TAU |
|  |  |  |  | Control | 76 | - | *Active:* Enhanced TAU (i.e., individualized care program including psychoeducation, psychological/ social/nursing therapies, and/or antidepressant medication) |

*Note*. ^a^Adapted from MBSR. ^b^Adapted from MBCT. ^c^Aggregate data was provided for analysis. ^d^Samples were coded as ‘physical health’ to differentiate them from substance use, depression, anxiety, and trauma despite potential overlap. SIM: self-reported interoception measure; M_age_: mean age; MBP: mindfulness-based programs; SIAD: sexual interest/arousal disorder; TAU: treatment as usual; MBCT: mindfulness-based cognitive therapy; MBSR: mindfulness-based stress reduction; MAIA: Multidimensional Assessment of Interoceptive Awareness; MAIA-TR: Trusting subscale of the MAIA; IBS: irritable bowel syndrome; VSI: Visceral Sensitivity Index; SBC: Scale of Body Connection; SBC-A: Awareness subscale of the SBC; SBC-D: Dissociation subscale of the SBC; CAQ: Cardiac Anxiety Questionnaire; BAQ: Body Awareness Questionnaire.

Table S2. Measures of psychological distress.

| **Measure Type** | **Measure Name** | **Measure Abbreviation** |
| --- | --- | --- |
| General | Brief Symptom Inventory | BSI |
|  | Depression Anxiety Stress Scales | DASS |
|  | Hospital Anxiety and Depression Scale | HADS |
|  | Kessler Psychological Distress Scale | K10 |
|  | Strengths and Difficulties Questionnaire | SDQ |
| Anxiety | Beck Anxiety Inventory | BAI |
|  | Behavioral Inhibition System – Anxiety | BIS-Anx |
|  | Brief Symptom Inventory – Anxiety Subscale | BSI-Anx |
|  | Depression Anxiety Stress Scales – Anxiety Subscale | DASS-Anxiety |
|  | Hospital Anxiety and Depression Scale – Anxiety Subscale | HADS-Anxiety |
|  | PROMIS Anxiety Scale | PROMIS-Anx |
|  | State-Trait Anxiety Inventory | STAI |
| Depression | Beck Depression Inventory | BDI |
|  | Brief Symptom Inventory – Depression Subscale | BSI-Dep |
|  | Behavioral Inhibition System –Depression | BIS-Dep |
|  | Center for Epidemiologic Studies – Depression Scale | CES-D |
|  | Depression Anxiety Stress Scales – Depression Subscale | DASS-Depression |
|  | Hospital Anxiety and Depression Scale – Depression Subscale | HADS-Depression |
|  | Hamilton Depression Rating Scale | HDRS or Ham-D |
|  | Patient Health Questionnaire | PHQ |
|  | PROMIS Depression Scale | PROMIS-Dep |
|  | Quick Inventory of Depressive Symptomatology | QIDS |
| Pain | Pain Catastrophizing Scale | PCS |
| PTSD | Crime-Related Post-Traumatic Stress Disorder Scale | CR-PTSD |
|  | PTSD Checklist for DSM-5 | PCL-5 |
| Stress | Depression Anxiety Stress Scales – Stress Subscale | DASS-Stress |
|  | Perceived Stress Scale | PSS |
| Other | Difficulties in Emotion Regulation Scale | DERS |
|  | Multidimensional Scale of Perceived Social Support | MSPSS |
|  | Dissociative Experiences Scale | DES |

Table S3. Measures of mindfulness.

| **Measure Name** | **Measure Abbreviation** |
| --- | --- |
| Five Facet Mindfulness Questionnaire | FFMQ |
| Freiburg Mindfulness Inventory | FMI |
| Mindful Attention Awareness Scale | MAAS |
| Philadelphia Mindfulness Scale | PHLMS |

Table S4. Literature correlations between SIMs for correlated-and-hierarchical effects models.

| Measure | MAIA_TOTAL | *MAIA_NO* | *MAIA_ND* | *MAIA_NW* | *MAIA_AR* | *MAIA_EA* | *MAIA_SR* | *MAIA_BL* | *MAIA_TR* | SBC_A | SBC_D | VSI | CAQ | BAQ |
| --- | --- | --- | --- | --- | --- | --- | --- | --- | --- | --- | --- | --- | --- | --- |
| MAIA_TOTAL | Correlation  (Source) |  |  |  |  |  |  |  |  |  |  |  |  | 0.56^a^ |
| *MAIA_NO* |  |  | 0.19^b^ | -0.02 | 0.43 | 0.58 | 0.38 | 0.45 | 0.21 |  |  |  |  |  |
| *MAIA_ND* |  |  |  | 0.08 | 0.16 | 0.13 | 0.17 | 0.18 | 0.15 |  |  |  |  |  |
| *MAIA_NW* |  |  |  |  | 0.24 | -0.05 | 0.2 | 0.01 | 0.22 |  |  |  |  |  |
| *MAIA_AR* |  |  |  |  |  | 0.38 | 0.62 | 0.48 | 0.42 |  |  |  |  |  |
| *MAIA_EA* |  |  |  |  |  |  | 0.46 | 0.56 | 0.21 |  |  |  |  |  |
| *MAIA_SR* |  |  |  |  |  |  |  | 0.55 | 0.43 |  |  |  |  |  |
| *MAIA_BL* |  |  |  |  |  |  |  |  | 0.33 |  |  |  |  |  |
| *MAIA_TR* |  |  |  |  |  |  |  |  |  |  |  |  |  |  |
| SBC_A |  |  |  |  |  |  |  |  |  |  |  |  |  |  |
| SBC_D |  |  |  |  |  |  |  |  |  | 0.175^c^ |  |  |  |  |
| VSI |  |  |  |  |  |  |  |  |  |  |  |  |  |  |
| CAQ |  |  |  |  |  |  |  |  |  |  |  |  |  |  |
| BAQ |  |  |  |  |  |  |  |  |  |  |  |  |  |  |

*Note*. All empty cells were imputed with 0.3 (the average correlation from [30]). Mindfulness and Psychological Distress correlations (within domains) were estimated at 0. ^a^[30]; ^b^[1]; ^c^average of [32]

Table S5. Risk of bias ratings.

|  | **Randomization** | **Blinding** | **Attrition** | **Outcome Bias** | **Reporting Bias** |  | **Overall Quality** |
| --- | --- | --- | --- | --- | --- | --- | --- |
| Bornemann et al., 2015 | 1 | 0 | 2 | 2 | 2 |  | 7 |
| Brotto et al., 2022 | 1 | 2 | 2 | 2 | 2 |  | 9 |
| Classen et al., 2021 | 1 | 0 | 2 | 2 | 2 |  | 7 |
| de Jong et al., 2016 | 2 | 0 | 2 | 1 | 2 |  | 7 |
| Duncan et al., 2017 | 2 | 1 | 2 | 2 | 2 |  | 9 |
| Fischer et al., 2022 | 1 | 1 | 2 | 2 | 2 |  | 8 |
| Fissler et al., 2016 | 2 | 1 | 2 | 2 | 2 |  | 9 |
| Francis et al., 2022 | 2 | 1 | 2 | 1 | 2 |  | 8 |
| Gawande, To, et al., 2019 | 1 | 0 | 2 | 2 | 2 |  | 7 |
| Gawande, Pine, et al., 2019 | 1 | 0 | 2 | 2 | 2 |  | 7 |
| Gawande et al., 2023 | 1 | 0 | 2 | 2 | 2 |  | 7 |
| Gaylord et al., 2011 | 2 | 1 | 2 | 1 | 2 |  | 8 |
| Henrich et al., 2020 | 1 | 0 | 2 | 2 | 2 |  | 7 |
| Karing & Beelmann, 2021 | 0 | 0 | 2 | 1 | 2 |  | 5 |
| Lima-Araújo et al., 2022 | 0 | 1 | 2 | 1 | 2 |  | 6 |
| Loucks et al., 2020 | 1 | 0 | 2 | 2 | 2 |  | 7 |
| Mittal et al., 2022 | 1 | 0 | 2 | 2 | 2 |  | 7 |
| Ugarte Pérez et al., 2023 | 1 | 1 | 2 | 0 | 2 |  | 6 |
| Price et al., 2005 | 1 | 1 | 2 | 2 | 2 |  | 8 |
| Price et al., 2012 | 1 | 0 | 2 | 2 | 2 |  | 7 |
| Price et al., 2019 | 1 | 0 | 2 | 1 | 2 |  | 6 |
| Price et al., 2020 | 1 | 0 | 2 | 2 | 0 |  | 5 |
| Price et al., 2023 | 1 | 0 | 1 | 2 | 2 |  | 6 |
| Roberts et al., 2021 | 2 | 0 | 2 | 1 | 0 |  | 5 |
| Segal et al., 2019 | 1 | 1 | 2 | 2 | 1 |  | 7 |
| Sharp et al., 2024 | 1 | 1 | 2 | 1 | 2 |  | 7 |
| Thomas et al., 2019 | 2 | 2 | 2 | 2 | 2 |  | 10 |
| van der Velden et al., 2023 | 2 | 0 | 2 | 2 | 2 |  | 8 |
| Vollbehr et al., 2022 | 2 | 0 | 2 | 1 | 2 |  | 7 |
| *Note.* Risk of bias was scored as follows: 2 = low risk of bias, 1 = some concerns, and 0 = high risk of bias. | | | | | | | |

Table S6. Moderation analyses for MBP interventions.

| **Name** | **Betas** | **CI-Low** | **CI-High** | | **P-values** | **FDR-Ps** |
| --- | --- | --- | --- | --- | --- | --- |
| Percent Female | 0.00 | -0.014 | 0.010 | 0.728 | | 0.989 |
| Mean Age | -0.02 | -0.032 | -0.006 | 0.019 | | 0.146 |
| MH Sample or Not | -0.08 | -0.407 | 0.244 | 0.571 | | 0.989 |
| MH Sample or Normative | -0.42 | -0.650 | -0.194 | 0.004 | | 0.062 |
| PH Sample Or Not | 0.00 | -2.101 | 2.092 | 0.989 | | 0.989 |
| PH Sample or Normative | -0.04 | -1.687 | 1.604 | 0.924 | | 0.989 |
| SampleSize | 0.00 | -0.002 | 0.005 | 0.163 | | 0.644 |
| Weeks Intervention | 0.01 | -0.348 | 0.359 | 0.917 | | 0.989 |
| Quality | -0.08 | -0.307 | 0.153 | 0.377 | | 0.942 |
| Practice Min_WOHome | 0.09 | -0.097 | 0.278 | 0.215 | | 0.644 |
| Practice MinWHome | 0.11 | -0.157 | 0.374 | 0.206 | | 0.644 |
| SessionAttendance | 0.42 | -4.787 | 5.623 | 0.774 | | 0.989 |
| Preregistered | 0.09 | -0.351 | 0.526 | 0.617 | | 0.989 |
| ControlType(Active) | 0.06 | -0.262 | 0.391 | 0.641 | | 0.989 |
| ControlType(MindBody) | 0.07 | -1.588 | 1.730 | 0.844 | | 0.989 |

*Note*. There is a trend level effect for mental health vs normative but the groups are imbalanced (13 MH vs 1 Normative), and the effect is unlikely to generalize.


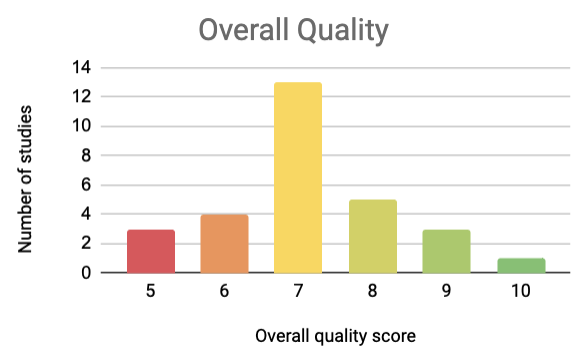


Figure S1. Overall quality of studies.


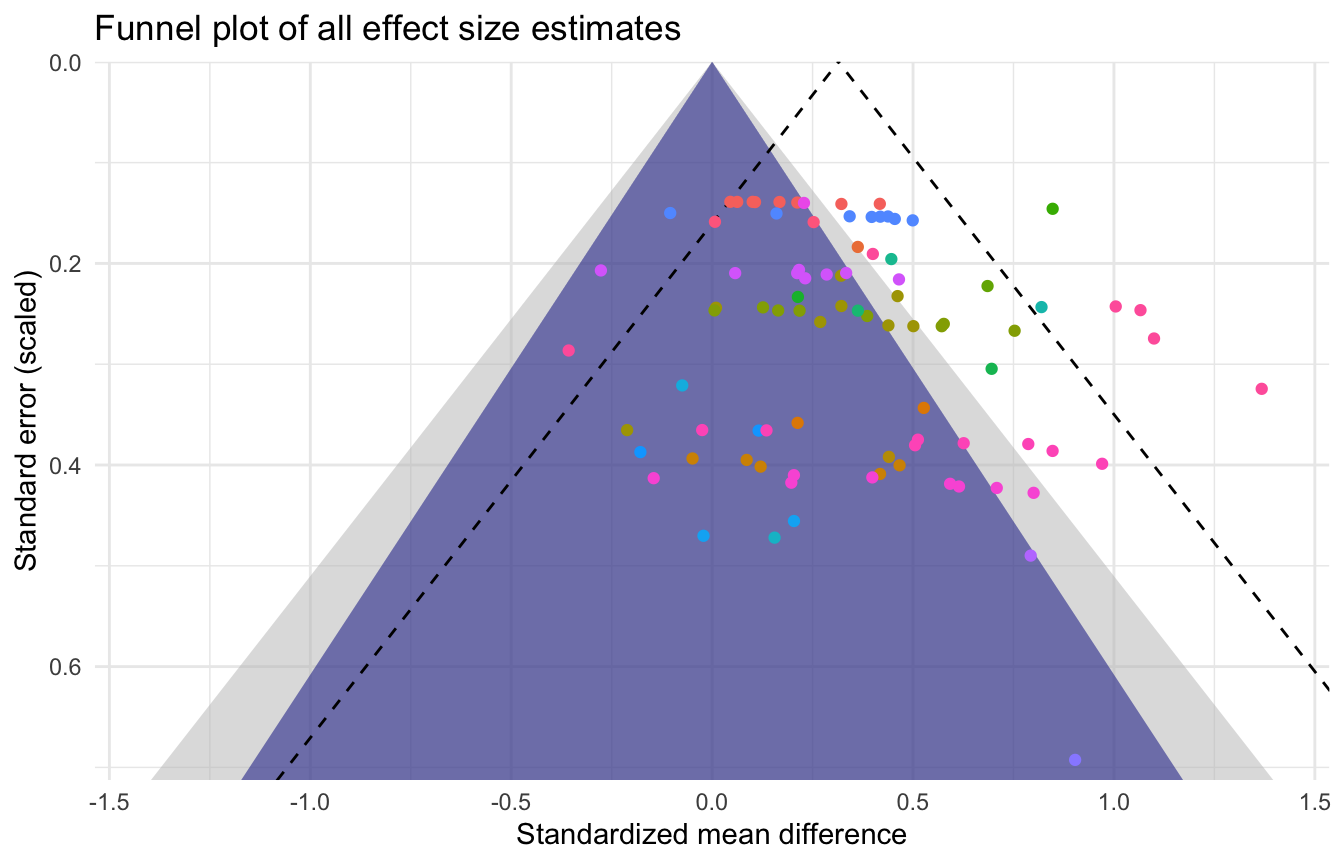


Figure S2: Funnel plot of all effect size estimates.

Studies with larger error variance (lower power) are expected to show more variable effect sizes. The displacement of the dotted funnel reflects the true effect compared to the null model (around a mean difference of 0). Publication bias is indicated by a bias towards positive mean differences for high standard error studies. Here is it shown that this bias occurs more often with lower SE studies, indicating larger effects for more well-powered studies. Colors: unique studies (an individual study may have multiple effects).


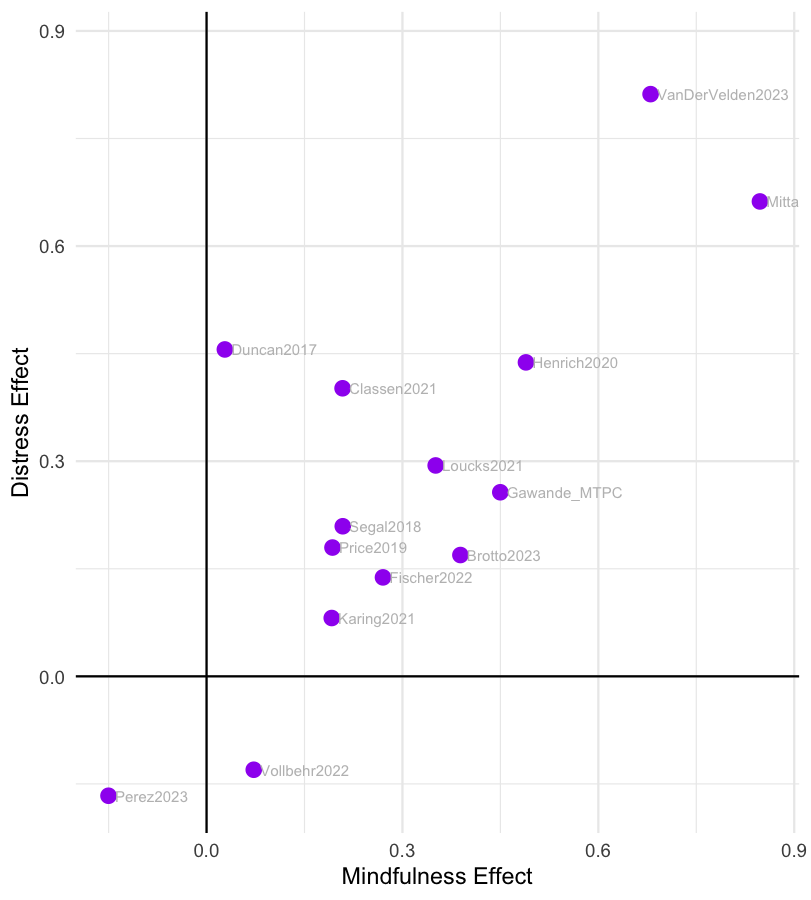


Figure S3. Changes in mindfulness vs changes in distress.

Each dot represents a study level effect, where positives represent improvements in mindfulness and decreases in distress.


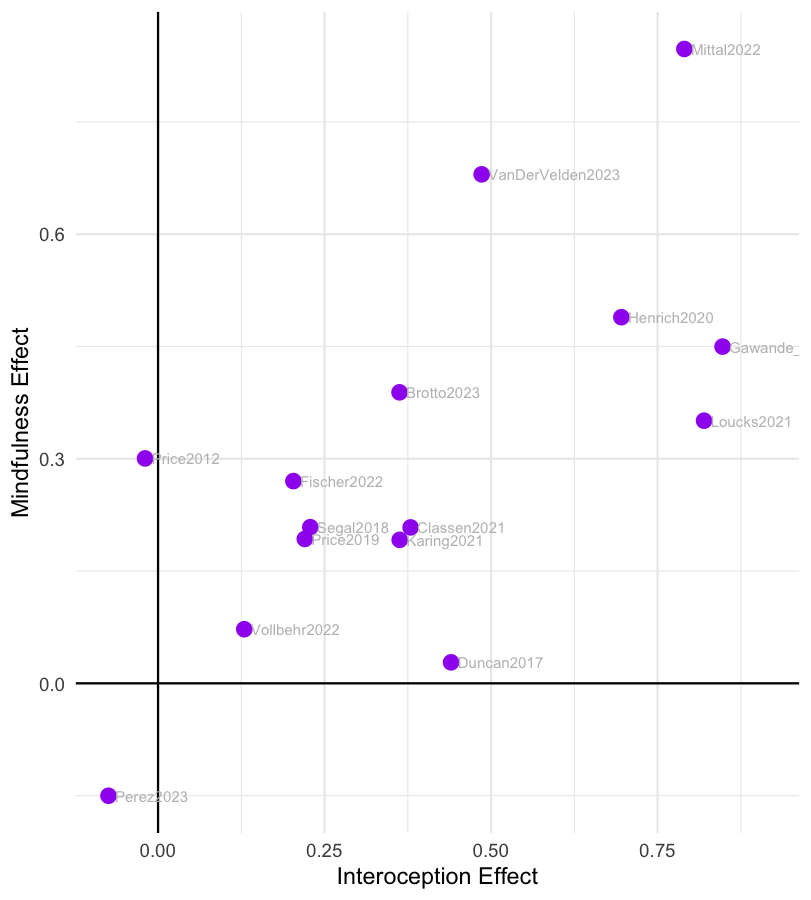


Figure S4. Changes in interoception vs changes in mindfulness.

Each dot represents a study level effect, where positives represent improvements in interoception and improvements in mindfulness.

References

1. Bornemann, B., Herbert, B. M., Mehling, W. E. & Singer, T. Differential changes in self-reported aspects of interoceptive awareness through 3 months of contemplative training. *Front. Psychol.* **Volume 5-2014**, (2015).

2. Brotto, L. *et al.* Mindfulness and Sex Education for Sexual Interest/Arousal Disorder: Mediators and Moderators of Treatment Outcome. *J. Sex Res.* **60**, 1–14 (2022).

3. Classen, C. C. *et al.* A Pilot RCT of A Body-Oriented Group Therapy For Complex Trauma Survivors: An Adaptation of Sensorimotor Psychotherapy. *J. Trauma Dissociation* **22**, 52–68 (2021).

4. de Jong, M. *et al.* Effects of Mindfulness-Based Cognitive Therapy on Body Awareness in Patients with Chronic Pain and Comorbid Depression. *Front. Psychol.* **Volume 7-2016**, (2016).

5. Duncan, L. *et al.* Benefits of preparing for childbirth with mindfulness training: A randomized controlled trial with active comparison. *BMC Pregnancy Childbirth* **17**, (2017).

6. Fischer, J. *et al.* Stress Reduction by Yoga versus Mindfulness Training in Adults Suffering from Distress: A Three-Armed Randomized Controlled Trial including Qualitative Interviews (RELAX Study). *J. Clin. Med.* **11**, (2022).

7. Fissler, M. *et al.* An Investigation of the Effects of Brief Mindfulness Training on Self-Reported Interoceptive Awareness, the Ability to Decenter, and Their Role in the Reduction of Depressive Symptoms. *Mindfulness* **7**, (2016).

8. Francis, S. E. B., Shawyer, F., Cayoun, B., Enticott, J. & Meadows, G. N. Group Mindfulness-Integrated Cognitive Behavior Therapy (MiCBT) Reduces Depression and Anxiety and Improves Flourishing in a Transdiagnostic Primary Care Sample Compared to Treatment-as-Usual: A Randomized Controlled Trial. *Front. Psychiatry* **Volume 13-2022**, (2022).

9. Gawande, R. *et al.* Mindfulness training enhances self-regulation and facilitates health behavior change for Primary Care patients: A randomized controlled trial. *J Gen Intern Med* **34**, 293–302 (2019).

10. Gawande, R. *et al.* Insurance-reimbursable mindfulness for safety-net Primary Care patients: A pilot randomized controlled trial. *Mindfulness N* **10**, 1744–1759 (2019).

11. Gawande, R. *et al.* Impact of Warm Mindfulness on Emotion Regulation: A Randomized Controlled Effectiveness Trial. *Health Psychol.* **42**, (2023).

12. Gaylord, S. *et al.* Mindfulness Training Reduces the Severity of Irritable Bowel Syndrome in Women: Results of a Randomized Controlled Trial. *Am. J. Gastroenterol.* **106**, 1678–88 (2011).

13. Henrich, J., Gjelsvik, B., Surawy, C., Evans, E. & Martin, M. A Randomized Clinical Trial of Mindfulness-Based Cognitive Therapy for Women With Irritable Bowel Syndrome—Effects and Mechanisms. *J. Consult. Clin. Psychol.* **88**, (2020).

14. Karing, C. & Beelmann, A. Evaluating the Implementation and Effectiveness of a Low-Dose Mindfulness-Based Intervention in a Student Sample: a Randomized Controlled Trial. *Mindfulness* **12**, (2021).

15. Lima-Araújo, G. *et al.* The impact of a brief mindfulness training on interoception: A randomized controlled trial. *PLOS ONE* **17**, e0273864 (2022).

16. Loucks, E. *et al.* Mindfulness-Based College: A Stage 1 Randomized Controlled Trial for University Student Well-Being. *Psychosom. Med.* **Publish Ahead of Print**, (2020).

17. Mittal, T. K. *et al.* Mindfulness-based intervention in patients with persistent pain in chest (MIPIC) of non-cardiac cause: a feasibility randomised control study. *Open Heart* **9**, e001970 (2022).

18. Ugarte Pérez, C. *et al.* Comparative efficacy of remotely delivered mindfulness-based eating awareness training versus behavioral-weight loss counseling during COVID-19. *Front Psychol* **14**, 1101120 (2023).

19. Price, C. Body-oriented therapy in recovery from child sexual abuse: an efficacy study. *Altern. Ther. Health Med.* **11**, 46–57 (2005).

20. Price, C. J., Wells, E. A., Donovan, D. M. & Rue, T. Mindful awareness in body-oriented therapy as an adjunct to women’s substance use disorder treatment: a pilot feasibility study. *J. Subst. Abuse Treat.* **43**, 94–107 (2012).

21. Price, C. J. *et al.* Immediate effects of interoceptive awareness training through Mindful Awareness in Body-oriented Therapy (MABT) for women in substance use disorder treatment. *Subst. Abuse* **40**, 102–115 (2019).

22. Price, C. J., Merrill, J. O., McCarty, R. L., Pike, K. C. & Tsui, J. I. A pilot study of mindful body awareness training as an adjunct to office-based medication treatment of opioid use disorder. *J. Subst. Abuse Treat.* **108**, 123–128 (2020).

23. Price, C. J., Sevinc, G. & Farb, N. A. S. Within-Person Modulation of Neural Networks following Interoceptive Awareness Training through Mindful Awareness in Body-Oriented Therapy (MABT): A Pilot Study. *Brain Sci.* **13**, 1396 (2023).

24. Roberts, R. L., Ledermann, K. & Garland, E. L. Mindfulness-oriented recovery enhancement improves negative emotion regulation among opioid-treated chronic pain patients by increasing interoceptive awareness. *J. Psychosom. Res.* **152**, 110677 (2021).

25. Segal, Z. V. *et al.* Practice of therapy acquired regulatory skills and depressive relapse/recurrence prophylaxis following cognitive therapy or mindfulness based cognitive therapy. *J. Consult. Clin. Psychol.* **87**, 161–170 (2019).

26. Sharp, M. *et al.* Prenatal Mindfulness Training and Interoceptive Awareness in Pregnant People at Risk for Hypertensive Disorders. *J. Integr. Complement. Med.* **30**, 1200–1208 (2024).

27. Thomas, E. A. *et al.* Mindfulness-Oriented Recovery Enhancement Restructures Reward Processing and Promotes Interoceptive Awareness in Overweight Cancer Survivors: Mechanistic Results From a Stage 1 Randomized Controlled Trial. *Integr. Cancer Ther.* **18**, 1534735419855138 (2019).

28. van der Velden, A. M. *et al.* Mindfulness Training Changes Brain Dynamics During Depressive Rumination: A Randomized Controlled Trial. *Biol. Psychiatry* **93**, 233–242 (2023).

29. Vollbehr, N. K. *et al.* Mindful yoga intervention as add-on to treatment as usual for young women with major depressive disorder: Results from a randomized controlled trial. *J. Consult. Clin. Psychol.* **90**, 925–941 (2022).

30. Desmedt, O., Heeren, A., Corneille, O. & Luminet, O. What do measures of self-report interoception measure? Insights from a systematic review, latent factor analysis, and network approach. *Biol. Psychol.* **169**, 108289 (2022).

31. Bornemann, B., Herbert, B. M., Mehling, W. E. & Singer, T. Differential changes in self-reported aspects of interoceptive awareness through 3 months of contemplative training. *Front. Psychol.* **5**, (2015).

32. Price, C. J., Thompson, E. A. & Cheng, S. C. Scale of Body Connection: A multi-sample construct validation study. *PLoS One* **12**, e0184757 (2017).
